# A Monte Carlo-Based 3D Whole Lung Model for Aerosol Deposition Studies: Implementation and Validation

**DOI:** 10.3390/bioengineering12101092

**Published:** 2025-10-10

**Authors:** Georgi Hristov Spasov, Ciro Cottini, Andrea Benassi

**Affiliations:** 1 Chiesi Farmaceutici S.p.A., 43122 Parma, Italy; 2 International School for Advanced Studies (SISSA), 34136 Trieste, Italy

**Keywords:** lung deposition, Monte Carlo simulation, whole lung models, orally inhaled drug products, therapeutic aerosols

## Abstract

A detailed picture of how an aerosol is transported and deposited in the self-affine bronchial tree structure of patients is fundamental to design and optimize orally inhaled drug products. This work describes a Monte Carlo-based statistical deposition model able to simulate aerosol transport and deposition in a 3D human bronchial tree. The model enables working with complex and realistic inhalation maneuvers including breath-holding and exhalation. It can run on fully stochastically generated bronchial trees as well as on those whose proximal airways are extracted from patient chest scans. However, at present, a mechanical breathing model is not explicitly included in our trees; their ventilation can be controlled by means of heuristic airflow splitting rules at bifurcations and by an alveolation index controlling the distal lung volume. Our formulation allows us to introduce different types of pathologies on the trees, both those altering their morphology (e.g., bronchiectasis and chronic obstructive pulmonary disease) and those impairing their function (e.g., interstitial lung diseases and emphysema). In this initial activity we describe deposition and ventilation models as well as the stochastic tree construction algorithm, and we validate them against total, regional, lobar, and sub-lobar deposition for healthy subjects.

## 1. Introduction

In addition to the treatment of several lung diseases, pulmonary drug delivery has recently been proposed as a root to administer compounds with systemic effects such as vaccines, gene therapies, insulin, and other small bio-molecules [1,2,3,4]. The administration of inhaled medicines relies on the production of aerosols of fine particles/droplets, their entrainment and transport in an air stream, and their deposition inside the patients bronchial tree. Understanding and mastering these phenomena is thus of paramount importance for improving design and optimization of orally inhaled drug products. In addition to pharmaceutical applications, the study of aerosol deposition in human lungs can be of great interest to those studying damages or diseases due to occupational exposure to toxic, pathogenic, or carcinogenic aerosols [5].

The description of the transport of a solid/liquid phase of various concentrations in an inhaled air stream can be a very complex task owing to the strong non-linearity of the air dynamics, the multi-scale nature of the aerosol–air interaction, and given the self-affine structure of the lungs themselves. The difficulty in measuring or imaging air and aerosol properties in vivo, i.e., in realistic physiological conditions, adds up to the complexity. In recent years, this has motivated the proliferation of modeling and simulation approaches of different natures: empirical or semi-empirical, fluid dynamics-based, and statistical. However, not all of them are accompanied by a comprehensive description of all the physical assumptions and numerical approximations adopted and, in many cases, they cannot be used to make quantitative predictions. In a recent series of works, we discuss how computational fluid dynamics (CFD) can fail its quantitative prediction of local aerosol deposition in human airways due to modeling assumptions and numerical approximations [6,7,8]. For example, while the computed total and lobar deposition appear to be independent from the spatial discretization, any local deposition estimation is found to be extremely sensitive to spatial discretization, with no clear convergence path with increasing resolution. Other major limitations are the difficulty turbulence models have in correctly capturing the turbulent-to-laminar transition that air experiences when penetrating deeper the bronchial tree, and the possibility to correctly account for the particle/droplet–turbulence interaction. Moreover, CFD simulations are usually limited to the first few upper airways (5–6th generation) extracted, through segmentation, from patient’s computed tomography scans (CT-scans). Any prediction on the fate of inhaled aerosols and on drug delivery efficacy, solely based on the amount of particles/droplets surviving deposition in these few proximal airways, is completely fictitious as it completely ignores the following: (i) changes in the airflow regimes, and role of air velocity fluctuations in the deeper airways; (ii) local changes in functional airway morphology or ventilation due to pathologies; (iii) mechanical deformation of the distal airways and alveoli during breathing; and iv) the possibility for the particles/droplets to be exhaled back in the atmosphere. The Stochastic Individual Path (SIP) approach attempts to overcome some of these issues [9,10,11]. It consists of a series of coupled multi-scale CFD simulations, each capturing a certain number of generations for an individual airway bifurcating multiple times from the trachea to the alveolus. With the need to keep the computational cost affordable, several approximations are introduced when coupling the different simulations, e.g., choosing a certain turbulence model or forcing a certain type of airflow or suppressing velocity fluctuations below a certain generation. To the best of our knowledge, a systematic study on how these assumptions impact local particle deposition has never been published. In a recent high performance supercomputing project, we have been able to capture, in a single Direct Numerical Simulation (DNS), an individual airway bifurcating 23 times during a steady inhalation [12]. As DNS does not require any *a priori* assumption on turbulence, this work allows us, for the first time, to see how turbulence and time fluctuations evolve proceeding deeper and deeper in the bronchial tree. We found that although turbulent fluctuations are confined to the upper airways only, flow splitting at each bifurcation has the potential to generate unsteady oscillations propagating downstream and ultimately cause flow rate fluctuations even in the deepest generations where the flow regime is entirely dominated by viscous dissipation (laminar, no secondary flows). These types of simulations can certainly be used to improve SIP approaches which remain, to date, the most advanced way of predicting aerosol deposition based on fluid dynamics simulations.

Statistical deposition models predict aerosol deposition based on certain deposition probabilities which are functions of both the aerosol properties and the local duct geometry [13]. Concerning the duct geometry properties, the simplest and oldest models adopt average data tabulated for each generation, like the early work by Yeh e Schum [14]. In this model the deposition probabilities are directly applied to the aerosol concentration within the ducts rather than on individual particles. The MPPD implementation improved the tree description by averaging the calculated deposition results over many different bronchial tree paths whose morphometric data are also tabulated [15,16]. The first Monte Carlo simulation, where stochastic trajectories are generated for individual particles/droplets, was originally proposed by Koblinger and Hofmann (KH) [17]. In their first calculations the airway geometrical properties were also stochastically generated for each particle trajectory: as long as a particle survives deposition, traveling duct after duct, the duct length, diameter, and orientation are randomly extracted *on-the-fly* from known morphometric distributions [18]. The larger the number of generated aerosol particle trajectories, the better sampled the morphometric statistical distributions are, and the more accurate the deposition predictions are, fully accounting for the asymmetry of bifurcations and anisotropy in the ventilation. Only recently, some authors started to compute deposition on 3D, pre-constructed, complete bronchial trees [19,20,21]. The advantages of working with pre-constructed bronchial trees rather than generating them *on-the-fly* are multiple:

- Realistic patient-specific bronchial trees can be constructed. Their proximal airways can be efficiently and automatically extracted from large CT-scan databases thanks to recently developed machine learning-based algorithms [22,23,24,25]. If the lobe or sub-lobe volumes can also be extracted from CT-scans, volume-filling algorithms can be adopted to stochastically grow the deeper airways within them [26,27,28,29,30]. Complete bronchial trees with 23 or more generations can be easily constructed with realistic shapes and physiologically sound morphometric statistical distributions.
- Pre-constructed bronchial trees can be coupled to mechanical breathing models. This allows us to obtain airflow distributions coherent with the tree geometries and to reproduce realistic inhomogeneous ventilation based on patient-specific lung function data [31,32,33,34,35].
- Mathematical models can be applied to healthy pre-constructed trees to simulate different types and severities of lung diseases. Geometric or functional modifications can be applied to specific ducts or distributed along the tree, and deposition simulations can reveal how drug delivery is impaired by the progression of a disease. Thanks to recent advances in functional respiratory imaging, the disease progression can be modeled in a realistic and patient-specific way [36].

In this work we propose a revised Monte Carlo deposition algorithm, extending the original KH algorithm, which computes deposition on pre-constructed, complete, patient-specific, and 3D bronchial trees. We also perform a first model validation comparing numerical predictions with measured data for total, regional, lobar, and sub-lobar aerosol deposition in healthy subjects. A critical analysis is carried out, highlighting the physical and numerical assumptions that might affect the model’s prediction capabilities. Finally, the next steps towards the possibility to predict therapeutic aerosol deposition in patient-specific, diseased bronchial trees will be listed and discussed.

## 2. Model Description and Code Implementation

This section contains a detailed description of the models and algorithms employed in our aerosol deposition calculations. Section 2.1 illustrates the tree growth procedure and concludes with a quantitative evaluation of its adherence to the known morphometric data. Section 2.2 contains details and considerations on the airflow splitting at bifurcations and the consequent emerging tree ventilation. The next three subsections are dedicated to the description of the deposition code algorithm as well as its input and output information. The adopted probability models are discussed in Section 2.3.4 while their equations are reported in Appendix D. The section concludes with a sensitivity analysis for the model parameters in Section 2.3.5, ensuring that the computed physical results are independent from the adopted numerical approximations.

### 2.1. Bronchial Tree Construction

**Figure 1:**
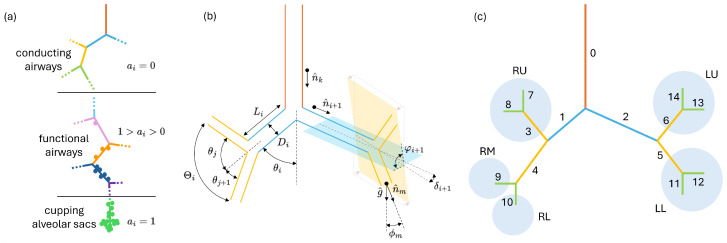
(**a**) Sketch of a single airway path highlighting its different partitions. (**b**) Sketch of a single bifurcation and geometry parameters characterizing its ducts. (**c**) Representation of the bronchial tree and its duct indexing convention, with index 0 labeling the trachea (RU right upper, RM right middle, RL right lower, LU left upper, and LL left lower lobes).

This section describes the bronchial trees adopted in the deposition simulations. They extend from the trachea, with duct index 0, to the capping alveaolr sacs at generation 23; a sketch of a typical airway path is presented in Figure 1a. The upper part of the bronchial tree is purely conductive, i.e., no alveoli are present to promote gas exchange with the blood in the circulatory system; an alveolation index $a_{i}$ can be defined and set to zero for the ducts of this portion of the tree. A transition to the functional airway usually happens between generations 12 and 15 where the first alveoli start to appear at the duct sidewalls; here $a_{i}$ starts to be greater than zero. The remaining part of the bronchial tree down to the final capping alveolar sac is called *acinus*; here $a_{i}$ grows progressively with the amount of alveoli populating the ducts until the terminal sac is reached and $a_{i}$ is set to 1. The choice of normalizing $a_{i}$ to 1, with $a_{i}=1$ only in the final capping alveolar sac, will prove to be useful for deposition calculations where it will be directly used as the probability for a depositing particle to enter an alveolus. From the perspective of the statistical deposition model, the bronchial tree is nothing but a collection of cylindrical ducts in series and parallel, determined solely by their length $L_{i}$, diameter $D_{i}$, branching angle $\theta_{i}$, gravity angle $\phi_{i}$, and alveolation index $a_{i}$. These are, in fact, the only geometry parameters entering the deposition probability equations. Figure 1b visualizes the different geometry parameters for a generic bifurcation. A bronchial tree is thus uniquely defined once the latter quantities are specified for every duct of the tree (The duct orientation versor $\hat{n_{i}}$ is stored in place of $\theta_{i}$ and $\phi_{i}$ as the two angles can be easily derived from it. In fact $\cos\left(\phi_{i}\right)=\hat{n_{i}}\;\cdot \;\hat{g}$ where $\hat{g}$ is the gravity versor (0,0,−1) and $\cos\left(\theta_{i}\right)=\hat{n_{i}}\;\cdot \;\hat{n_{k}}$ where *k* is the parent duct of *i*.) and a convention for the numbering of the ducts is chosen. The indexing convention adopted hereafter is illustrated in panel (c) of Figure 1; in the hypothesis of dichotomous branching, the connectivity of the tree, i.e., which children are connected to which parent, is easily derived from the simple algebraic equations detailed in Appendix A.

For the considerations and the validation presented in this first work, we rely on a bronchial tree generated with a stochastic growth algorithm very similar to the one described by KH in their pioneering work on Monte Carlo aerosol deposition simulation [17]. Their stochastic algorithm allows the bronchial tree to grow with no spatial constraints: length, diameter, and orientation of ducts are all extracted from statistical distribution and correlations they derived from morphometric measurements [18]. As suggested by KH, the first 4–5 generations should not be grown through the stochastic algorithm; the value of their geometry parameters should be obtained by tabulated data of real patients. The geometry of the proximal airways is in fact extremely subject-specific and KH had not enough statistics to make sure their distributions are reliable below generation 6. For the present analysis we extracted the upper airway geometry parameters from subjects of the public computed tomography (CT) scan repository ATM’22 [24]. Coming from patient-specific medical imaging data, the non-planarity angle $\delta_{i}$ can be slightly different from zero and the rotation angle $\varphi_{i}$ can be different from π/2 for this proximal tract of the tree. In the subsequent stochastically generated part of the bronchial tree, $\delta_{i}=0$ and $\varphi_{i}=\pi /2$ by construction. Following KH, the first 12 generations of the tree are purely conductive, i.e., $a_{i}=0$. After generation 12 a probability function is interrogated, by extracting a random number in the interval [0,1], to decide whether the airway remains conducting or becomes functional (Figure 4 of reference [18] and analytical equation therein). In the latter case, the following is set:

(1) $$a_{i}=\frac{z}{12}-\frac{11}{12},$$

with *z* being the generation to which the *i*-th duct belongs. This rule ensures that, at generation z=23 when the terminal alveolar sac is met, $a_{i}=1$. Imposing the correct average generation number at which conducting airways switch to functional ones is important to correctly match the density of acini estimated through microscopy studies. KH estimated a number of acini between 20 and 48 thousands [18]; Hansen and Appaya gave the value 23 thousands based on their measurements [37]; and estimations from other authors are collected by Montesantos et al. [28] ranging from 23 to 66 thousands. Our bronchial trees have roughly 36 thousands acini, a value in agreement with the one by KH and of the same order of magnitude of those proposed by other authors.

Based on the morphometric analysis of a single acinus by Haefeli-Bleuer and Weibel [38], KH have foreseen the possibility of a premature termination of the acinus with a capping alveolar sac before generation 23. Although realistic, the premature capping of the acini gives rise to a significant underestimation of the number of alveolar sacs if coupled with the assumption of a maximum of 23 generations in the tree. With the idea of maintaining a maximum of 23 generations in our trees, a choice dictated by the need of keeping the computational cost and the code complexity low, we show in Appendix B that a good solution is represented by terminating all the airways at generation 23 without enforcing premature capping. Trees generated in this way ensure a correct number of alveolar sacs (around eight million) and, at the same time, represent an acceptable approximation of physiological trees extending beyond the 23-rd generation and being only *on average* terminated at generation 23.

In the conducting airways the diameters $D_{i}$ and $D_{i+1}$ of the two children ducts in a branch are determined recursively from the diameter of the parent duct $D_{m}$:

(2) $$D_{i}=\frac{D_{m}\;r_{2}}{\sqrt{r_{1}\left(r_{2}^{2}+1\right)}}\;D_{i+1}=\frac{D_{m}}{\sqrt{r_{1}\left(r_{2}^{2}+1\right)}},$$

where $r_{1}$ and $r_{2}$ are two random numbers extracted from the KH distributions for the ratio of parent-to-children cross section area (Figure 7 of reference [18]) and for the ratio of minor-to-major child (Figure 8 of reference [18]), respectively. Concerning the duct length $L_{i}$, a correlation exists with the diameter $D_{i}$; the solution proposed by Kitaoka et al. [39] has been preferred in place of the KH correlation due to its ease of implementation: $L_{i}=D_{i}\;r_{3}$, where $r_{3}$ is a random number extracted from a normal distribution with mean 2.8 and standard deviation 1.0. The lack of morphometric information for the distal airways induced KH to perform a simplification for the functional airways (acini): any asymmetry in the children ducts is suppressed and the child length and diameter scaling laws are simplified [17]. In particular, for the length they have chosen $L_{i}=D_{i}\;2.2$ based on the data by Haefeli-Bleuer et al. [38]; their choice for the diameter scaling is not clear so a new law has been defined, directly fitting the dataset by Haefeli-Bleuer et al., where $D_{i}$ is related to the parent duct diameter $D_{m}$ by the following:

(3) $$D_{i}=D_{m}\;2^{-1/8.93}.$$

**Figure 2:**
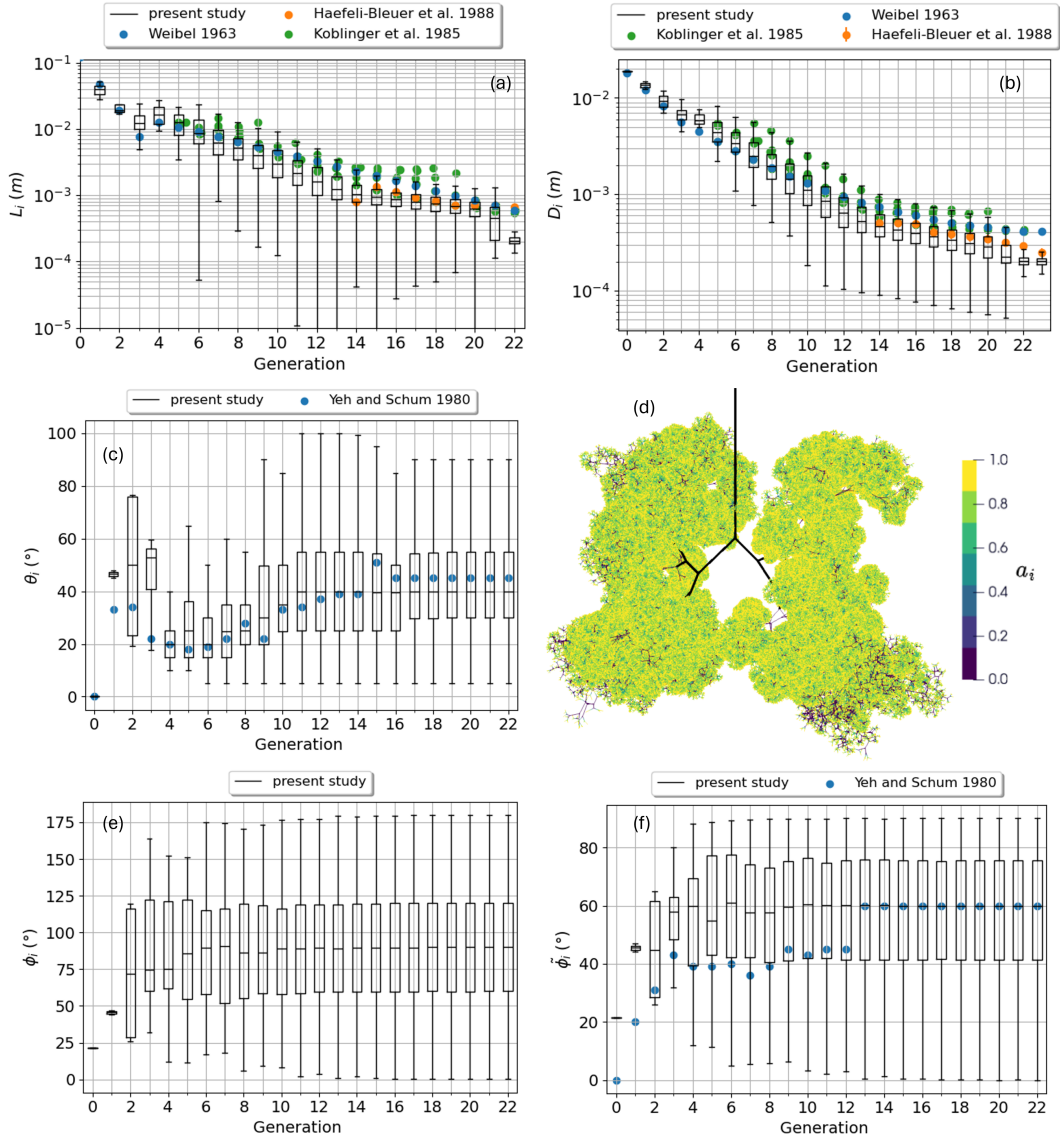
Box plots for (**a**) duct length, (**b**) duct diameter, and (**c**) branching angle as a function of the generation number for a KH-like tree. (**d**) Three-dimensional representation of a KH-like tree with 23 generations. The color code shows the alveolation index distribution among the ducts. The thick black line highlight the first 4 generations extracted from a CT-scan. Box plots for (**e**) gravity angle and (**f**) KH-corrected gravity angle, according to Equation (A5). When available, average data measured by different authors are overlaid for comparison [14,18,38,41].

Once a particle enters an alveolus, the only geometry parameter necessary to compute the deposition parameter is the alveolus diameter $D_{i}$. For both the side alveoli and those belonging to the capping alveolar sac terminating the airway, we extract $D_{i}$ from a Gaussian distribution with mean 200.17μm and standard deviation 19μm, based on the measurements and the statistics by Ochs et al. [40]. The box plots of panels (a) and (b) of Figure 2 show the statistics for length and diameter of ducts across the generations (The statistics shown in the panels of Figure 2 is collected on a single tree; the variability among different trees and different tree growth algorithms will be the subject of a forthcoming dedicated work). Experimental datasets have been overlaid for comparison, including the Weibel model A dataset [41], the acinus data by Haefeli-Bleuer and Weibel [38], and the single subject series employed by KH to extract their distributions [18]. The expected change in slope, when switching from conducting to functional airways, is visible for both $L_{i}$ and $D_{i}$. Our average results tend to slightly underestimate both lengths and diameters if compared to the Weibel ones; however, the discrepancy is comparable with the inter-subject variability of the KH series and with the discrepancy between the Weibel model and the Haefeli-Bleuer data for the acinar region. We can thus conclude that the average properties of our tree are in agreement with the existent literature and that the variability within and between available datasets makes any further refinement meaningless.

In the conducting airways the branching angle $\theta_{i}$, i.e., the angle between the directions of the parent and child ducts, is correlated to the child duct diameter. In particular, in each bifurcation it is possible to define a major and a minor child based on their duct diameters. KH defined two different statistical distributions to sample from for the attribution of major and minor branching angles (Figure 13 of reference [18]). More precisely, KH presented cumulative distribution functions $F_{z}^{ma}\left(\theta \right),\;F_{z}^{mi}\left(\theta \right)$, giving the probability to find a branching angle below θ for a duct pertaining to the *z*-th generation. After digitalizing and interpolating their distributions, the probability distribution functions $f_{z}^{ma}\left(\theta \right),\;f_{z}^{mi}\left(\theta \right)$ have been computed and normalized to be used to extract a random value for $\theta_{i}$ for each duct based on its generation index *z*. The distributions presented by KH cover only generations from 6 to 19. For all the others below and above this interval, one of the two extremes have been used (this situation occurs rarely in our trees as generations below the 6th are typically extracted from patient-specific CT-scans rather than being generated randomly, while for those greater than the 12th the switch to functional airway changes the generation rules.). When the airway becomes functional and the children asymmetry disappears, we use the major distribution only for both the branches. This rule differs from the one chosen by KH using, as distribution, the average between major and minor of the highest generation, i.e., $[f_{19}^{ma}\left(\theta \right)+f_{19}^{mi}\left(\theta \right)]/2$. The impact of this different choice is negligible; in fact, as KH point out [17], for z≥16 one has $f_{z}^{ma}\left(\theta \right)≃f_{z}^{mi}\left(\theta \right)$ and both are almost independent from *z*. The statistics for $\theta_{i}$ as a function of the generation number is shown in the box plot of Figure 2c. The agreement with the measurements by Yeh and Schum [14] is remarkable.

This unconstrained growth model does not allow us to directly control the shape of the tree nor the orientation of its ducts with respect to gravity; a picture of the tree is shown in panel (d) of Figure 2. KH recognized that by averaging the gravity angle over all the ducts of each distal generation, a value of ≃90° was found, i.e., an equal number of ducts is pointing upward and downward so that the average orientation results would be horizontal. They proposed a correction to the duct orientation to slightly bias them downward and obtain an average gravity angle of ≃60° in agreement with the experimental observation on lung casts by Yeh and Schum [14]. In Appendix B we demonstrate this correction is not necessary as the average value of ≃60° comes from a rescaling adopted by Yeh and Schum in the definition of the gravity angle itself and it corresponds to ≃90° with the standard, and more intuitive, definition of gravity angle adopted by KH and in this work. The box plot in Figure 2e displays the statistics of the gravity angle $\phi_{i}$ as a function of the generation number according to the convention adopted in this paper, while in panel (f) the angles have been transformed according to Equation (A6) to allow a direct comparison with the dataset by Yeh and Schum. Indeed, for distal airways the agreement is very good and in line with the observation of KH of an equal distribution of upward and downward oriented ducts.

### 2.2. Airflow Behavior in the Bronchial Tree

Aiming at predicting the aerosol deposition along the airways, the average airflow rate $Q_{i}$ of each duct, or its average air velocity $U_{i}=Q_{i}/\left(\pi /4D_{i}^{2}\right)$, must be computed. No other property of the airflow enters the deposition probability equations, as it will be illustrated in Section 2.3. If an inhalation profile or a tidal breathing need to be simulated, $Q_{0}$ and thus all the $Q_{i}$ become functions of time, assuming positive values during inhalation phases and negative ones during exhalation; $Q_{0}=Q_{i}=U_{i}=0$ during breath-holding maneuvers. The $Q_{i}$ should be, in principle, computed by setting the pressures at the terminal part of the bronchial tree, e.g., at the alveolar sacs, and calculating all the pressure drops $\Delta p_{i}$ across the ducts knowing their resistance. The simplest way to compute the resistance of a duct is through the Hagen–Poiseuille equation. However, this neglects the secondary flow generated at the bifurcation and assumes the airflow to be laminar. Corrections have been proposed by Pedley et al. [42,43]; together with an explanation on why their modified equation is expected to hold even in the presence of turbulence, further improvements are also available [44]. On the other hand, the pressure at the alveolar sacs can be set by coupling this ventilation model to a set of equations describing the breathing mechanics through the stiffness/compliance of the lung tissues. Some examples exist in the literature [31,32,33,34,35], although in most of them the bronchial tree is terminated at the beginning of the functional airways with the whole acinus accounted implicitly by the mechanical breathing model. Moreover, while enhancing the quality of the airflow description, these ventilation models substantially increase the computational cost and the complexity of the code, especially considering that the $Q_{i}$ values should be updated at every time-step.

For this first critical analysis and validation work, we prefer to rely on an empirical approach to compute the $Q_{i}$, similar to the one adopted by KH. They recognize that, at each bifurcation, the airflow splitting between the two children depends on the change in volume occurring downstream each of them which, on its part, is proportional to the total alveolar volume downstream each of them $v_{i}$. In this view two splitting coefficients $s_{i}$ and $s_{i+1}$ for the two children can be defined according to the following:

(4) $$s_{i}=\frac{v_{i}}{v_{i}+v_{i+1}}\;s_{i+1}=\frac{v_{i+1}}{v_{i}+v_{i+1}},$$

and used to compute the flow rate in the children ducts, given the parent flow rate $Q_{m}$, as follows:

(5) $$Q_{i}=s_{i}\;Q_{m}\;Q_{i+1}=s_{i+1}\;Q_{m}.$$

**Figure 3:**
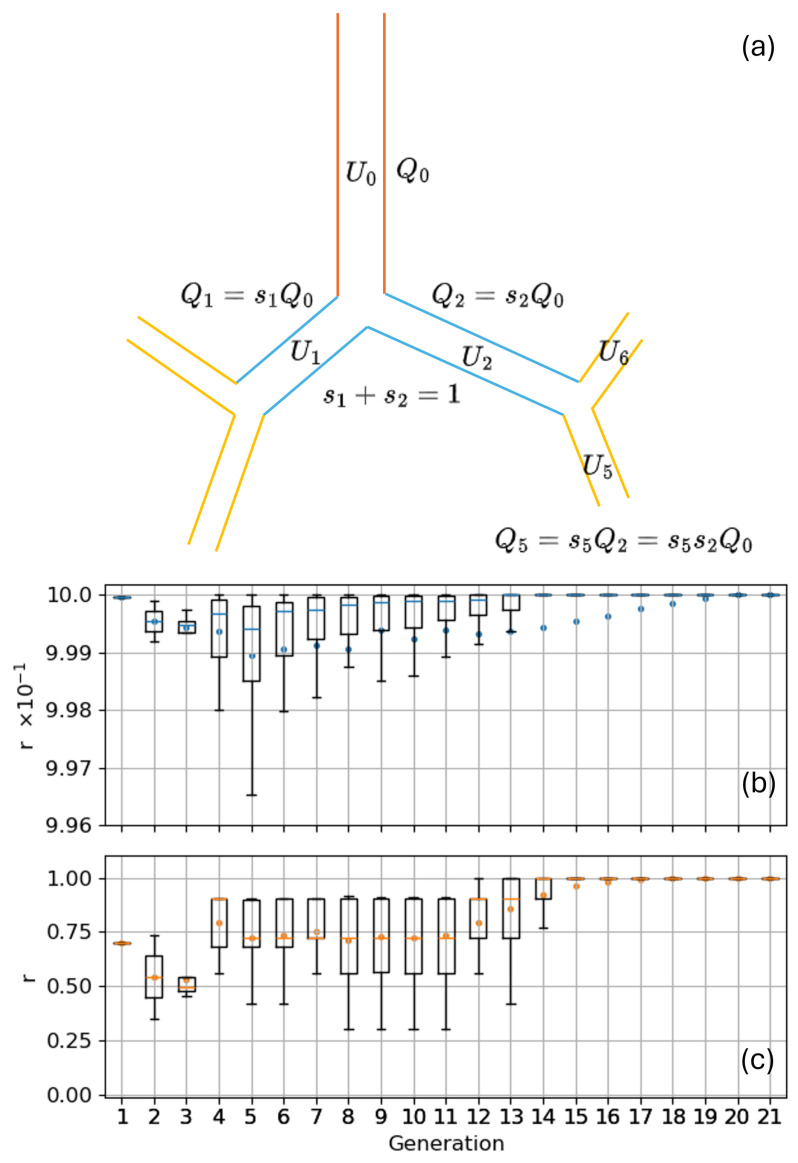
(**a**) Sketch of the airflow splitting procedure and definition of splitting coefficients. Box plots of the flow splitting asymmetry ratio as a function of the generation number for (**b**) splitting rule (4) and (**c**) splitting rule (9).

Notice how, by construction, $s_{i}+s_{i+1}=1$ ensures the flow rate conservation; this airflow splitting procedure is schematized in Figure 3a. The alveolar volume downstream each duct $v_{i}$ can be computed through the alveolation index $a_{i}$, knowing the total lung volume $V_{tot}$ that must be provided as an imput parameter (e.g., say 5.8 L for an average adult healthy male), in a three steps procedure as follows:

- the total volume of the ducts $V_{ducts}$ can be computed, after the bronchial tree is generated, as follows:
  (6) $$V_{ducts}=\sum_{i=1}^{N_{tot}}v_{i}=\sum_{i=1}^{N_{tot}}\frac{\pi }{4}D_{i}^{2}\;L_{i},$$
  where the volume of each individual duct $v_{i}$ has been assumed cylindrical;
- subtracting the duct volume to the total lung volume one can compute the total alveolar volume $V_{alv}=V_{tot}-V_{ducts}$;
- $V_{alv}$ must now be distributed in the alveolated ducts based on their local alveolation index $a_{i}$, to this aim one can define which is the fraction of the total alveolar volume corresponding to each $a_{i}$ value as follows:
  (7) $$v_{i}=a_{i}\;V_{alv}/\left(\sum_{j=1}^{N_{tot}}a_{j}\right).$$
  Now the total alveolar volume downstream the *i*-th duct can be computed as follows:
  (8) $$v_{i}=\sum_{j\in t_{i}}f_{j}\;v_{j},$$
  with $t_{i}$ subset of all the ducts downstream the *i*-th one, i.e., the sub-tree generated from the *i*-th duct. $f_{j}$ is a functional factor representing the efficiency of the alveoli if the *j*-th duct to stretch and recall air. $f_{j}$ can be used to represent diffuse or localized losses of lung function due to diseases (e.g., fibrosis or emphysema) or to account for a different local deformation of the alveoli due to a different elasticity/stretchability of parenchyma. According to Equation (8) ventilation anisotropy can thus be induced in a tree by acting on both $a_{j}$, i.e., on the local alveolar density, and $f_{j}$. Being interested in modeling homogeneous and healthy lungs, unless explicitly stated, in all the calculations presented in this work $f_{j}$ is set to one for all the ducts of the bronchial trees.

KH, however, do not use a fixed bronchial tree geometry; they generate individual airways along with each particle trajectory, and thus are unable to quantify the $v_{i}$ based on the above described procedure. This is why they decided to impose a flow splitting proportional to the cross-sectional area of the two daughters as follows:

(9) $$s_{i}=\frac{D_{i}^{2}}{D_{i}^{2}+D_{i+1}^{2}}\;s_{i+1}=\frac{D_{i+1}^{2}}{D_{i}^{2}+D_{i+1}^{2}}.$$

Both definitions (4) and (9) are insufficient to capture the correct flow splitting inside the bronchial tree. Indeed, Equation (4) allows us to control the ventilation anisotropy or to simulate functional disease by modulating the distribution of alveoli $a_{i}$ or their functionality $f_{i}$. In a healthy bronchial tree, however, where the distribution of alveoli in the distal airways is rather uniform, $s_{i}≃s_{i+1}≃0.5$ causes a symmetric splitting of the flow rate between left and right lobes (and sub-lobes). This non-physiological condition is visualized in the box plot of Figure 3b, where the flow splitting asymmetry ratio $r=max\left\{Q_{i},Q_{i+1}\right\}/min\left\{Q_{i},Q_{i+1}\right\}$ is plotted for each generation, and is caused by the absence of any local geometry parameter in Equation (4). Conversely, Equation (9) depends only on the local geometry parameters of the children ducts and is thus able to promote the expected asymmetry in the airflow splitting for the proximal bifurcations. It also allows us to account for diseases altering the local duct morphometry, e.g., bronchiectasis or asthma. However, the splitting is not influenced at all by the distribution/functionality of alveoli in the downstream portion of the tree, making the model unsuitable to simulate functional diseases. A reasonable compromise is achieved by mixing the two splitting hypotheses:

(10) $$s_{i}=\frac{D_{i}^{2}\;v_{i}}{D_{i}^{2}\;v_{i}+D_{i+1}^{2}v_{i+1}}\;s_{i+1}=\frac{D_{i+1}^{2}\;v_{i+1}}{D_{i}^{2}\;v_{i}+D_{i+1}^{2}v_{i+1}},$$

i.e., we combined the splitting model based on the downstream volume only with the one based on the local geometry parameters only. Notice how, if the bifurcation is perfectly symmetric, $D_{i}^{2}≃D_{i+1}^{2}$ and Equation (4) are readily obtained; conversely, if the alveolar distribution/functionality is uniform inside the sub-trees, $v_{i}≃v_{i+1}$, and one is left with Equation (9). If the previous conditions are met, $s_{i}=s_{i+1}=0.5$ and the tree ventilation is homogeneous. Figure 3c shows the flow splitting asymmetry ratio *r* when rule (10) is adopted; the asymmetry in the flow splitting induced by the local geometry parameters is clearly visible for the upper airways. Notably the splitting between the left and right lobe is 42–58% in agreement with measured values of 46–54% [45]. It is important to stress that, for healthy subjects, the adoption of Equation (10) rather than (4) is found to alter the deposited fraction prediction by less than 3–4%. As it will be shown in Section 3, this variation is much smaller than the typical inter-subject variability.

Starting from the *i*-th duct, and applying the flow splitting rules multiple times for all the upstream bifurcations until the trachea is reached, one is left with the product of the splitting coefficients for all the ducts upstream of the *i*-th one:

(11) $$Q_{i}=\left(\prod_{j\in w_{i}}s_{j}\right)\;Q_{0}=S_{i}\;Q_{0},$$

where $w_{i}$ denotes the set of all the ducts along the airway path leading to the *i*-th one and $S_{i}$ is the cumulative splitting coefficient. Knowing all the $S_{i}$ of a bronchial tree allows us to compute the average velocity in each duct for any possible flow rate $Q_{0}$. This is particularly advantageous in case of time dependent flow rates when $Q_{0}$ changes continuously over time but the $S_{i}$ are computed only once for a given tree. Clearly the present model does not contemplate a flow rate-dependent airflow splitting.

### 2.3. Aerosol Transport and Deposition

This section details the structure of the aerosol transport and deposition algorithm as well as its assumptions and approximations. A first subsection describes the input information necessary to characterize the tree ventilation and the aerosol physical and statistical properties. Next a description follows on how the aerosol particle/droplet trajectories evolved differently depending on the breathing phase. A third subsection discusses how the output can be processed and analyzed. The selected models for the deposition probability calculation are listed in the fourth subsection alongside their implications and their adaptation to the different lung regions and breathing phases. A final subsection presents a sensitivity study for the main model parameters.

#### 2.3.1. Input Parameters

To quantify the aerosol deposition in each duct/alveolus, it is necessary to know its set of geometry parameters ($D_{i}$, $L_{i}$, $\theta_{i}$, and $\phi_{i}$), and the average air velocity $U_{i}$ flowing through it. The latter is estimated from the flow rate at the mouth/nose/trachea $Q_{0}$ according to the procedure illustrated in Section 2.2. With the interest in studying bronchial deposition consequent to ambient aerosol breathing, or due to the administration of a therapeutic aerosol through an inhaler, the flow rate is typically a function of time, i.e., $Q_{0}=Q_{0}\left(t\right)$, representing a periodic tidal breathing or a single transient inspiratory maneuver. As all the aerosol deposition datasets we use for model validation are obtained using tidal breathing conditions, in the present work we set the following:

(12) $$Q_{0}\left(t\right)=\frac{\pi \;V_{tidal}}{T}sin\left(\frac{2\pi }{T}t\right),$$

with *T* breathing period, $V_{tidal}$ tidal volume, and the overall duration of the deposition simulation $T_{tot}$ being a multiple of *T*. In deriving the equations for the deposition probabilities, $U_{i}$ and $Q_{i}$ are assumed constant, and the inhalation profile $Q_{0}\left(t\right)$ is thus transformed into a stepped function with step size Δt: within each Δt interval, $Q_{0}$ is constant and equal to its average value in the interval itself. Clearly Δt must be small enough that the stepped function remains able to capture all the representative features of the inhalation profile, including fast variations if present. Moreover, this discretization is purely numerical, the deposition predictions can be considered meaningful only if they are independent from the choice of Δt; in this respect a sensitivity analysis has been performed and its results illustrated in Section 2.3.5.

**Figure 4:**
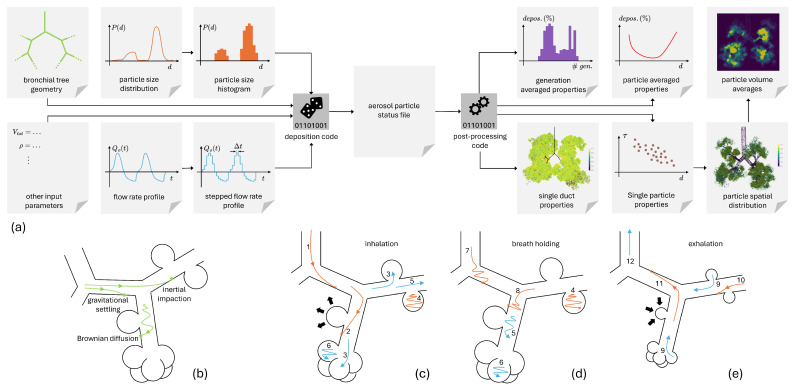
(**a**) Sketch of inputs and outputs of the deposition code. (**b**) Sketch of the three mechanisms promoting aerosol deposition in the bronchial tree. (**c**–**e**) Possible different behaviors of aerosol particles/droplets during inhalation, breath-holding, and exhalation phases according to the current implementation of the code.

Particles are assumed to be spherical and are characterized by their diameter *d* and by their density ρ. A poly-disperse aerosol can be modeled if its particle size number distribution $P_{N}\left(d\right)$ is provided. Our Monte Carlo algorithm is based on the generation of single particle trajectories; we thus need to rely on number distributions, as typical particle size analyzers (based on laser light scattering or on image analysis) measure mass distributions $P_{M}\left(d\right)$. One can easily switch between the two distributions by the transformation $P_{M}\left(d\right)=P_{N}\left(d\right)\;\rho \;\pi d^{3}/6$. If available as a continuum function, $P_{N}\left(d\right)$ is discretized and the particle diameters of the aerosol cloud are randomly extracted from the generated probability histogram. In this first validation work we concentrate on the calculation of deposition fractions, so we considered only uniform particle size distributions from 10 nm to 10μm, i.e., the number of particles per diameter $N_{ppd}$ released in the bronchial tree is the same for every diameter. The set of input parameters is illustrated on the left side of Figure 4a.

#### 2.3.2. Algorithm Description

As illustrated in panel (b) of Figure 4, there are three competing mechanisms leading to aerosol particle/droplet deposition in the bronchial tree: inertial impaction, for large particles in the bifurcations of the upper airways; settling, for those particles surviving inertial impaction at bifurcations but still heavy enough to be influenced by gravity; and Brownian diffusion, chaotically disturbing the trajectory of small particles and promoting collisions on the distal airway/alveoli walls Finlay [46]. The probability equations for these mechanisms are detailed in Section 2.3.4, together with their underlying assumptions, as well as oropharyngeal and nasal deposition probabilities. There are also the possible breathing phases contemplated by our model, and they are illustrated in panels (c)–(e) of Figure 4; their duration is indicated as $\Delta T_{i}$, $\Delta T_{bh}$ and $\Delta T_{e}$ for inhalation, breath-holding, and exhalation, respectively.

During inhalation $Q_{0}$ is positive, and aerosol can enter the patient’s airways: for every particle of density ρ and diameter *d* extracted from the P(d) histogram, an initial insertion time $T_{start}$ is randomly extracted within a custom insertion window $\Delta T_{ins}$. In this early validation work $\Delta T_{ins}$ coincides with the whole inhalation duration $\Delta T_{i}=T/2$; the aerosols of interest are in fact administered by gentle and slow nebulization in tidal breathing conditions. More generally, different types of inhalers can release their therapeutic dose over different ranges of duration, from $\Delta T_{ins}∼$ tens of milliseconds to a few seconds in dry powder inhalers; $\Delta T_{ins}$ = 300–400 ms in pressurized metered dose inhalers. The flow rate at the insertion instant $Q_{0}\left(T_{start}\right)$ is computed and used within the nasal/oral deposition probability equation to determine whether the particle survives or deposits within the extra-thoracic airways. The same $Q_{0}\left(T_{start}\right)$ value is used to guess if the particle survives the trachea; in case of positive outcome, a particle flight time τ is defined as $L_{0}/U_{0}$, i.e., the time taken by the particle to cross the trachea. For every subsequent *i*-th duct, in case of survival, τ is incremented by adding the corresponding crossing time $\tau_{i}=L_{i}/U_{i}$. In the same way, before computing the new deposition probability for the *i*-th duct, the flow rate at the mouth must be updated using $Q_{0}\left(\tau \right)$, and the local flow rate $Q_{i}$ re-computed using Equation (11). Naturally, this procedure is meaningful only if, during the duct crossing time $L_{i}/U_{i}$, $Q_{0}$ and thus $Q_{i}$ and $U_{i}$ remain reasonably constant. This is not always the case. In fact $U_{i}$ can be very small either in the very distal airways, where the $Q_{i}$ are small in consequence of the flow splitting among a large number of pipes, or because $Q_{0}\left(t\right)$ is approaching zero at the end of an inhalation phase. In both cases the crossing time might be extremely long and, meanwhile, $Q_{0}\left(t\right)$ can change significantly. Such an unphysical situation can be avoided by modifying the algorithm proposed by KH, introducing the stepped flow rate profile and its time-step Δt. If $L_{i}/U_{i}\leq \Delta t$ the flow rate remains practically constant during the time it takes the particle to cross the *i*-th duct, the KH algorithm above described applies. In the opposite case the particle equation of motion for the axial coordinate along the duct x(t):

(13) $$\frac{dx(t)}{dt}=U_{i}\left(t\right)=\frac{4}{\pi D_{i}^{2}}\;Q_{i}\left(t\right)=\frac{4S_{i}}{\pi D_{i}^{2}}\;Q_{0}\left(t\right)$$

is integrated by means of an implicit Euler algorithm (see Appendix C) until $x\left(t^{★}\right)=L_{i}$ or $x(t^{★})=0$, i.e., until the particle reaches one of the two ends of the ducts at time $t^{★}$. This means $t^{★}$ is the new crossing time and it is necessary to set $\tau_{i}=t^{★}$ before computing the deposition probability. It can take a time greater than T/2 or even greater than *T* for the particle to reach one of the duct ends, in which case $U_{i}\left(t\right)$ changes signs towards the different inhalation and exhalation phases, i.e., the particle can move back and forth many times along the duct axis.

When a particle survives deposition, successfully crossing a parent duct, it must progress downstream to be assigned to one of the two children, the choice can be made in different ways: (i) in a symmetrical fashion, with 50% probability to enter in each child; (ii) with an asymmetric probability based on some geometry parameter, e.g., the children cross-sectional area ratio; and (iii) with an asymmetric probability based on the airflow splitting. The latter has been implemented in our code being the most realistic and making use of the local splitting coefficient already computed through Equation (10). A random number $r_{5}$ is extracted in the interval [0,1] and the particle is assigned to the *i*-th child if $r_{5}\leq s_{i}$ or to the (i+1)-th child if $r_{5}>s_{i}=1-s_{i+1}$.

The possible fate of a particle reaching the bronchial tree during an inhalation phase is sketched in Figure 4c:

- A particle can deposit in a conducting airway (1).
- A particle can deposit in a functional airway. A random number $r_{4}$ is extracted in the interval [0,1] and compared to the local alveolation index $a_{i}$ which, being normalized to 1, works as the probability to enter an alveolus. If $r_{4}>a_{i}$ the particle deposits on the airway wall (2), otherwise it enters a local alveolus or a capping alveolar sac (3).
- Once in an alveolus a particle can deposit therein driven by settling or diffusion (4), no inertial impaction is contemplated as the air velocity is supposed to be negligible.
- Lastly, at the end of an inhalation phase, a particle can still be traveling in a conducting or functional duct (5) or floating in an alveouls (6).

During breath-holding phases aerosol insertion is suspended, as $Q_{0}=Q_{i}=U_{i}=0$, only settling and diffusion are at work to cause particle/droplet deposition in the current duct/alveolus. For particles ending the inhalation phase in conditions (5) and (6), the time spent in the last duct/alveolus must be summed up with the total breath-holding time $\Delta T_{bh}$. With this new $\tau_{i}$ the deposition probabilities must be recalculated and the chance of deposition must be evaluated again. The possible phenomenology is captured by Figure 4d:

- A particle starting in condition (5) can deposit in a conducting (7) or a functional (8) duct. In the latter case the same procedure described for condition (2) applies.
- A particle starting in condition (6) can deposit in the alveolus like in (4).
- As for inhalation, particles can still survive the breath-holding phase remaining suspended in a duct (5) or in an alveolus (6).

It is important to note that breath-holding is an optional phase separating an inhalation from an exhalation; in our code it can be avoided by setting $\Delta T_{bh}=0$. Being primarily concerned with tidal breathing, in most of the deposition simulations presented here, breath-holding is absent. Unless explicitly stated it must be assumed $\Delta T_{bh}=0$; the impact of the breath-holding maneuver on therapeutic aerosol deposition will be evaluated extensively in a dedicated future work.

Also during an exhalation phase, aerosol insertion is suspended. Moreover all the particles trapped in the alveoli, condition (6), are released as a consequence of the alveoli contraction, condition (9) in Figure 4e. These particles, together with those of conditions (5) and (6), are now free to travel through the ducts in the upstream direction. When a particle survives deposition crossing the *i*-th duct it is automatically assigned to the parent duct whose index is readily obtained via Equation (A4). Again, during an exhalation phase,

- All particles are expelled from the alveoli, as in condition (9). Particles within a capping alveolar sac will start the exhalation phase in its parent duct; particles within a side alveolus will be re-assigned to the corresponding functional duct.
- A particle traveling the airways can deposit in conducting (11) or functional (10) ducts. In the latter case the same procedure described for condition (2) applies.
- A particle can survive until the next inhalation phase, in which case the conditions of Figure 4c apply again. Or, in its ascent, a particle can escape the trachea, condition (12), and reach the extra-thoracic airways. Another attempt for particle deposition is made against the nasal or oropharyngeal probability functions, in case of failure the particle is eventually exhaled in the atmosphere.

#### 2.3.3. Output Analysis

When a particle deposits, information on its flight time τ, diameter, and deposition place (duct index or alveolus type) are stored in an *aerosol status file* to be analyzed later, producing statistics. Several random numbers are also extracted to generate fictitious deposition coordinates on the cylindrical duct surface. In fact a 3D binning of the deposition coordinates leads to a deposition density map comparable to those coming from lung scintigraphy and single photon emission computed tomography. Clearly, the comparison makes sense only as long as the duct is smaller or comparable to the size of the bins. For proximal airways it can be misleading as the latter are in fact large in size and dominated by inertial impaction which is characterized by very localized deposition patterns.

The aerosol status file also stores information on exhaled particles and on those particles still traveling in the bronchial tree at time $T_{tot}$, i.e., when the simulation ends. Different post-processing tools read the aerosol status file and extract several types of information, as sketched on the left side of Figure 4a. The most extractable aggregated data are the aerosol fractions deposited in the whole bronchial tree and the exhaled fractions. Generation averaged or diameter averaged quantities follow, e.g., the fraction of aerosol deposited in each generation or the total deposition fraction per diameter. On the opposite side one has the single duct properties, e.g., fraction of particle deposited in the *i*-th duct and its particle size distribution. At the same level, single particle properties can also be processed and correlated with each other, e.g., particle residence time and its correlation with the particle diameter, correlation between particle diameter, and the generation of deposition. Plotting the particle deposition place, through the assigned fictitious coordinates, generates a 3D cloud of points visualizing the density of aerosol deposition or other scalar properties associated with single particles. Through a 3D binning of the deposition coordinates, a local aerosol density can also be computed, as well as volume averaged particle properties (the volume average being on the 3D bin/voxel).

#### 2.3.4. Aerosol Deposition Probabilities

This section offers a quick overview of the particle deposition probabilities implemented in our code. Readers interested in the specific models adopted, their equations, and details about their implementation are referred to Appendix D.

The deposition in the extra-thoracic airways, nose, or mouth–throat tract is computed according to the deposition efficiency curves recently proposed by Cheng and fitted from in vitro deposition data [47]. They predict particle deposition based on the flow rate $Q_{0}$ and the aerodynamic diameter $d_{ae}=d\sqrt{\rho /\rho_{w}}$ with $\rho_{w}=1000$ kg/m^3^ water density. According to Cheng the adopted model is adequate to describe extra-thoracic deposition in both inhalation and exhalation phases.

For the intra-thoracic deposition several models are available; most of them require different equations for deposition in ducts or alveoli, depending on the specific breathing phase. The inertial impaction probability $I_{i}$ is dominant for large and heavy particles, stopping them in the upper airways. Many empirical equations have been proposed over the years for $I_{i}$, and most of them have been summarized by Finlay [46]. Some equations for $I_{i}$ depend on the particle Stokes number only, while others include a dependence on the branching angle $\theta_{i}$, such as the one by Yeh and Schum [14] adopted in this work. The probability of deposition by gravitational settling $G_{i}$ is relevant for heavy or large particles in small cross section ducts, i.e., in the distal airways. The rationale to compute it stems from the comparison of the time $\tau_{i}$ it takes for a particle to cross the duct length $L_{i}$ and the time necessary for the same particle to meet a duct wall while moving along the gravity direction (e.g., for a horizontal duct the time taken to travel a distance $D_{i}$ under the action of gravity). Also, for this probability we adopt the formulation originally proposed by Yeh and Schum [14] and subsequently by KH. Finally, we also follow the same authors for the probability of deposition by Brownian diffusion $B_{i}$. Brownian diffusion impacts light or small size particles and is relevant for ducts of small cross section. The probability $B_{i}$ is computed by comparing the time $\tau_{i}$ it takes for a particle to cross the duct length $L_{i}$ and the time necessary for a diffusing particle to cross the duct width $D_{i}$, which depends on the particle diffusion coefficient in air $D_{c}$.

The probability for a particle to cross the *i*-th duct without depositing there, $P_{i}$, can be calculated as follows:

(14) $$P_{i}=\left(1-I_{i}\right)\left(1-G_{i}\right)\left(1-B_{i}\right)$$

i.e., considering the survival of the three different deposition mechanisms as independent events, this is the choice of several other authors [17,48,49,50]. Others have noticed that whilst each aerosol particle trajectory is ruled by an equation of motion, the three deposition mechanisms should not be considered independent and therefore have adopted the general form:

(15) $$P_{i}=1-{\left(I_{i}^{k}+G_{i}^{k}+B_{i}^{k}\right)}^{1/k}$$

with *k* taking different integer or fractional values in different models or, within the same model, in different regions of the bronchial tree [46]. However, such choice remains arbitrary and does not bind $P_{i}$ to the interval [0,1] as it should be appropriate for a probability function. In his book Finlay demonstrates how the deposition probability, calculated for different aerosol size and in different generations, remains relatively independent from the chosen *k*, except around *d* = 1 μm and in the deepest generations, which is, however, precisely the scenario of interest for therapeutic aerosols. Moreover, one substantial difference between our model and the original KH is that we consider, as a deposition unit, the individual ducts while KH consider the bifurcations. They estimate the local deposition in each bifurcation by mixing the probabilities of its parent and children ducts. To the best of our knowledge, no systematic analysis exists in the literature on how the choice of the $P_{i}$ equations, and their possible combination at bifurcations, affect particle deposition. Such analysis is beyond the scope of the present work and will be the subject of a dedicated forthcoming paper.

#### 2.3.5. Model Sensitivity Analysis

**Figure 5:**
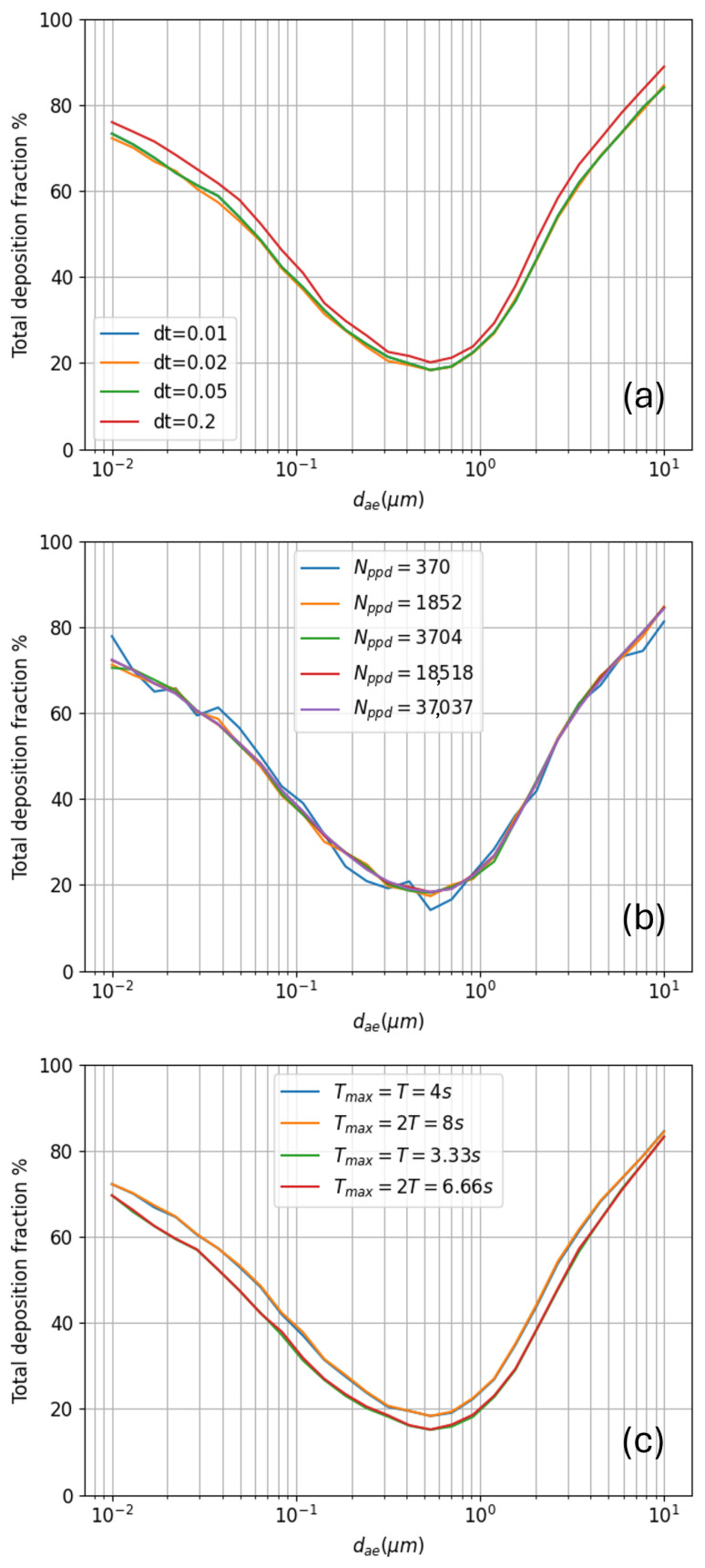
(**a**) Aerosol deposition fraction as a function of the aerodynamic diameter $d_{ae}$ for decreasing time-step Δt. (**b**) Aerosol deposition fraction as a function of the aerodynamic diameter $d_{ae}$ for increasing number of injected particles per diameter $N_{ppd}$. (**c**) Aerosol deposition fraction as a function of the aerodynamic diameter $d_{ae}$ for different tidal breathing period *T* and total simulation duration $T_{tot}$.

Among the model parameters the time-step Δt is certainly the most critical: as with any other simulation technique such as molecular dynamics or CFD, the physical results should be independent from the chosen temporal discretization, which is just a mathematical artifact. This is typically demonstrated by showing that the results of interest converge to stable values for Δt→0. Figure 5a shows how the total deposited fraction as a function of the aerosol size becomes independent of Δt if the latter takes values ≤50 ms. The deposition curves are obtained in tidal breathing mode with $T_{tot}=8$ s, $V_{tidal}=500$ mL, and T=4 s (corresponding to 15 breaths per minute), injecting a uniform distribution of particles with a number of particles per diameter $N_{ppd}$ =18,518 (a total of 0.5 milion particles) and density ρ=1000 kg/m^3^. The convergence with decreasing Δt ensures the time integration of particle trajectories described by Equation (13) and detailed in Appendix C is physically sound and does not spuriously alter the deposition statistics. A value Δt=20 ms has been selected for all the simulations presented in the following.

In our Monte Carlo approach all the information concerning global and local deposition is of statistical nature. To make sure the statistics is at proper convergence, a number of particles/droplets large enough must be injected for each diameter. In this way particles of all sizes have the possibility to travel the whole bronchial tree, in all its generations if physically possible, and to take a chance to deposit there. A convergence study for the deposition fraction as a function of the number of injected particles per diameter $N_{p}pd$ is presented in panel (b) of Figure 5; all the simulation parameters are the same as in panel (a) and Δt=20 ms. When increasing $N_{ppd}$ the initially noisy deposition curves smoothen and converge to a final shape independent of the number of injected particles. A value $N_{ppd}$ = 18,518 has been selected for all the simulations presented in the following.

The last point of attention, in case of tidal breathing, concerns the number of breathing cycles to be included in the simulations, i.e., the total simulation duration $T_{tot}$. Panel (c) of Figure 5 shows the aerosol deposition as a function of particle diameter, in tidal breathing conditions, where the aerosol is injected only during the first inhalation phase, i.e., in the interval [0,T/2]=[0,2s]. After terminating the simulation at $T_{tot}=4$ s the 0.33% of injected particles are still suspended in the bronchial tree, neither deposited nor exhaled (blue curve). One can add an entire breathing cycle, doubling $T_{tot}$, to give them enough time to face their fate (yellow curve). In this case only the 0.05% of particles is still found suspended in the tree at the end of the simulation. In both cases, however, the fraction of aerosol which remains suspended is so small that the deposition curves are practically superimposed. Naturally, changing the tidal breathing period *T* can result in variations of deposited fraction (green curve); however, as before, enhancing $T_{tot}$ to include more breathing cycles (red curve) has no effect at all on the deposition curves. A decrease in total deposited fraction with decreasing breathing period *T*, as the one presented in panel (c), has already been experimentally observed by Darquenne [51].

To conclude, for all the simulations presented in the following, a single breathing cycle is considered, and a check is always performed to make sure the residue of aerosol particles still suspended at the end of the simulation remains negligible. The effect of changing tidal volume and breathing period *T* on total, regional and local deposition will be analyzed in detail in the next sections.

## 3. Model Validation

Before taking a deep dive into a quantitative comparison of the model prediction with the existing experimental data on in vivo particle deposition, this section opens with a qualitative description of the aerosol behavior. This preliminary discussion serves both to illustrate how the results for the single particle trajectory/history are physically sound, and to highlight which kind of statistical information can be extracted from the simulations besides the deposition prediction.

**Figure 6:**
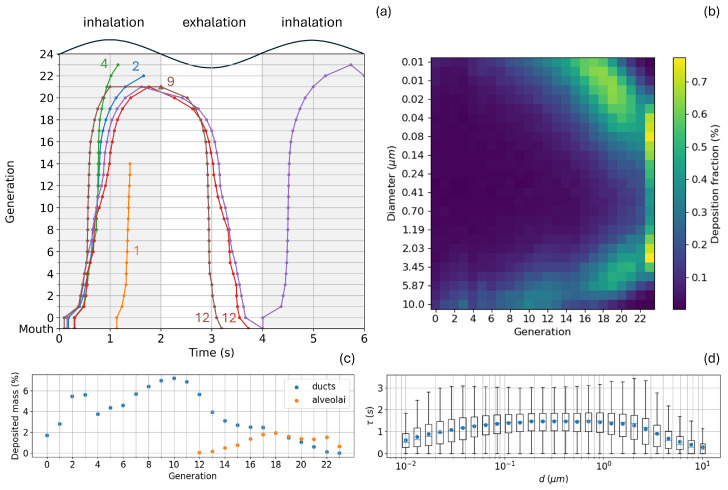
(**a**) Examples of aerosol particle trajectories, i.e., generation occupied by a particle as a function of time, during tidal breathing cycles. Numbers refer to the possible cases discussed in Section 2.3.2 and Figure 4. The inhalation phases are highlighted by a gray shade. (**b**) Color map representing the aerosol deposition fraction as a function of both the particle size and the generation number. (**c**) Deposited mass of aerosol as a function of the generation number distinguishing between deposition on duct walls of conducting or functional airways (blue), and inside side-wise alveoli of terminal alveolar sacs (yellow). (**d**) Box plot showing the correlation between particle flight time τ and diameter.

Figure 6a visualizes some example of particle trajectories during tidal breathing cycles with $V_{total}$ = 0.5 L, T=4 s, $T_{tot}=8$ s and uniformly distributed particles of density ρ=1000 kg/m^3^ in the range 10 nm to 10μm. The aerosol is injected only in the first inhalation phase (first 2 ms); as time increases, all the particles surviving deposition escalate their generation number. The yellow trajectory indicates a large particle sticking early in a conducting airway, as in case (1) according to the numbering of Section 2.3.2 and Figure 4. The blue and green trajectories represent smaller particles reaching the functional airways but facing a different fate once there: the former depositing on a duct wall, case (2); the latter depositing into an alveolus, case (4). The red trajectory particle is still traveling in a functional airway when the exhalation phase starts. It reverts its trajectory ascending the bronchial tree reaching the mouth and being exhaled, as in case (12). Another example of exhalation is represented by the brown trajectory. Here the particle succeeded in entering an alveolus but its residence time is not enough to permit its deposition. When exhalation starts the particle is expelled from the alveolus, case (9), and de-escalates its generation number until reaching the mouth. The violet trajectory highlights how particles with the right diameter minimizing the deposition probability, can travel back and forth in the bronchial tree multiple times. The color map of panel (b) displays the deposition profile along the generations for aerosol particles/droplets of different size. As expected, there are 10μm particle deposits already in the first generations (bottom left corner) mainly due to inertial impaction. At generation 8 to 10 Brownian motion starts to impact in small particles and the top part of the plot takes a green-yellow color. After generation 10, reading the map along the vertical axis, one can clearly see the characteristic *U*-shaped trend for particle deposition as a function of diameter already plotted in Figure 5. The bottom right corner is dark blue as no large particles can reach the deeper generations; the top right corner is also dark blue as some small particles are deposited along the way to the bottom of the bronchial tree, and those reaching there are easily exhaled back. Panel (c) of Figure 6 shows a typical pattern of deposition for a given inhaled dose through the generations. If and at which generation a maximum exists is strictly dependent on the injected aerosol distribution shape P(d). What is interesting to note here is that, from generation 18 onwards, the dose mass deposited in the alveoli is comparable to the one deposited along the duct walls. In other words, delivering the aerosol to the deepest generations does not ensure the particles will land directly inside the alveoli; a ∼50% chance exists for them to deposit on a duct wall near the alveolus entrance. Although in our model the alveoli geometry and their way of populating ducts are very idealized, we believe this fact remains valid and deserves deeper investigation through more realistic models and geometries. What will be called the *pulmonary* deposition in the following section considers all the dose mass deposited in the alveolated ducts, regardless of whether the deposition site is an alveolus or a duct wall. Lastly, the box plot of panel (d) illustrates the correlation between particle diameter and flight time; for particles of intermediate size, τ has an average value between 1.5 and 2 s with a large dispersion. A sudden drop of τ is expected for large particles due to the immediate action of inertial impaction in the proximal airways; a gentler drop is also seen for very small particles due to Brownian diffusion that scatters them, promoting their deposition all along the bronchial tree.

### 3.1. Total Deposition

**Figure 7:**
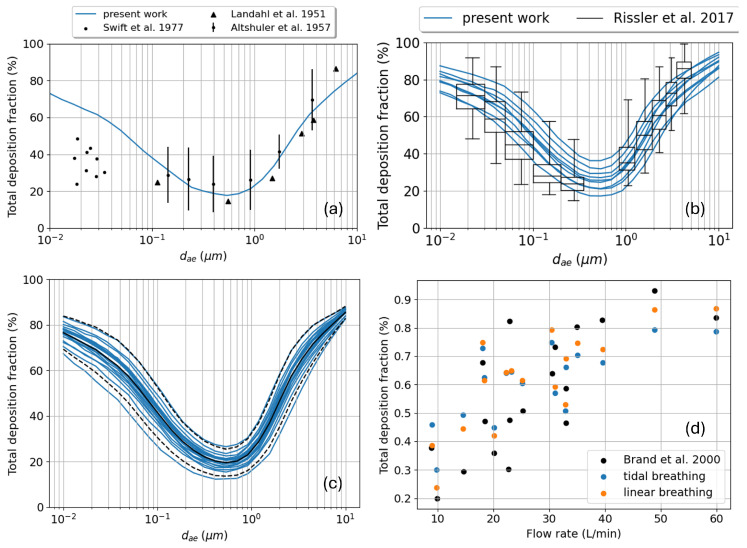
(**a**) Measured (black markers, from references [52,53,54]) and predicted (blue line) deposition fraction as a function of the aerodynamic diameter of the aerosol particles/droplets. (**b**) Measured (black box plot, from reference [55]) and predicted (blue lines) inter-subject variability in the deposition fraction as a function of the aerodynamic diameter. (**c**) Variability in the predicted deposition fraction as a function of the aerodynamic diameter for different stochastically generated bronchial trees. Each blue curve represents the total deposition fraction for single bronchial tree, the black continuous curve marks the average deposition trend while the two dashed curves represent the 95% confidence interval. (**d**) Measured (black dots, from reference [56]) and predicted (colored dots) deposition fraction as a function of the inhalation flow rate.

The total aerosol deposition as a function of the particle size in tidal breathing conditions is computed assuming the same parameters of Figure 6a. The resulting characteristic *U*-shaped curve is plotted in blue in Figure 7a against three referenced in vivo data sets [52,53,54]. Within the large inter-subject variability and variety of breathing conditions, the agreement is quite good, especially considering that our model does not use adjustable parameters such as the *enhancement factor* or the *mixing factor* introduced by KH [17]. A certain discrepancy is visible only for very small particle diameters, with the model overestimating deposition even by 20%. This suggests some correction is needed for the deposition probability by Brownian diffusion. A more recent dataset by Rissler et al. [55], reporting the variability in tidal breathing conditions among patients, allows us to analyze the model sensitivity to such parameters and to compare the resulting variability in aerosol deposition. They report minimum, mean, and maximum values for the tidal volume $V_{tidal}$ (480, 750, and 1060 mL) and breathing period *T* (3.4, 6, and 8.6 s) for the pool of analyzed subjects; nine different inhalation profiles can thus be constructed by combining this information. The comparison for the total deposition fraction is reported in Figure 7b; the box plot highlights the scatter in the experimental data, while the family of blue curves the scatter in the numerical simulations. As both are around 12–14% the two variabilities are definitely comparable in magnitude. Notice also how the deposition for small diameters measured by Rissler et al. is much higher than in the datasets of panel (a) making the match with the numerical prediction very satisfactory also in this range of sizes. Unfortunately, some major detail is missing in the description of the old dataset by Swift; thus, it is not possible to explain the discrepancy with the more recent one by Rissler. So far only the model sensitivity to the breathing conditions has been tested, but what about the sensitivity to the adopted tree geometry? To answer this question, we generated 20 different bronchial trees based on the procedure illustrated in Section 2.1, adopting different random seeds for the stochastic growth algorithm and different proximal airway geometries extracted from the scans of the ATM’22 repository [24]. The particle deposition fraction computed for these trees with the same breathing conditions of Figure 7a are reported in panel (c). The variability seems slightly smaller than the one related to the inhalation profile, with an 8% value. Clearly, the two variability ranges discussed in panels (b) and (c) do not sum up; in fact, in reality, each inhalation profile is tightly correlated to a specific lung morphology and lung function.

Brand et al. [56] investigated the behavior of the total deposition as a function of the flow rate magnitude; in Figure 7d the predictions of our model are compared with their dataset. For each involved subject, the authors recorded the inhaled volume (from 250 to 2000 mL) and the flow rate (from 10 to 60 L/min), without specifying the exact inhalation profiles. We thus constructed both a sinusoidal and a linear breathing pattern (In the latter case the inhaled volume is a piecewise linear function in time while the flow rate is a piecewise constant function in time.) and computed the total deposition for both. The trend of growing deposition with increasing flow rate is clearly confirmed; the scatter of the data also has the same amplitude of the experimental one as both the simulated breathing patterns. This confirms the capability of our model to correctly capture the inter-subject variability.

**Figure 8:**
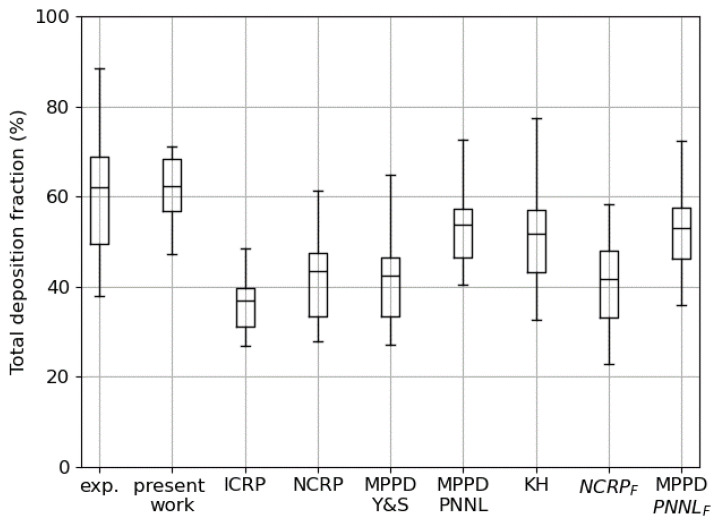
Comparison of the predictivity of total deposited fraction for our model and those widely adopted in the literature; plot adapted from reference [57]. MPPD has been run with both Yeh & Schum and PNNL morphometric data for the bronchial tree. Both the NCRP and MPPD models have also been corrected to account for the functional parameters; last two columns with *F* subscript.

In a recently published work Rissler et al. [57] compared their measured value of total deposited fraction over a wide population of subjects with the prediction of different semi-empirical models, such as the NCRP and ICRP ones [58,59], or stochastic tree-based models such as MPPD [15,16] or the reference work by KH. This work offers us the possibility to compare the predictivity of our new model against the widely adopted standard methods for deposition and pharmacokinetics studies. From the tabulated data it is possible to estimate an inter-subject variability for the tidal breathing parameters of $V_{tidal}=1.09\pm 0.37$ L and T=7.43±3.2 s for males and $V_{tidal}=0.78\pm 0.22$ L and T=5.58±1.74 s for females. As for the previously described comparison, a good representation of the variability in the deposition prediction is obtained by combining minimum, mean, and maximum values of both $V_{tidal}$ and *T*, obtaining nine different profiles and deposition curves per sex. The total deposition fraction is then obtained by averaging over male and female results, exactly as it has been done for the data recorded on patients. The comparison is shown in Figure 8: our prediction is the closest to the experimental value; the reduced variability is probably due to the fact that all the calculations have been performed on the same bronchial tree, and running more simulations over a wide pool of stochastically generated trees might increase it; the semi-empirical models significantly underestimate deposition, as well as the MPPD one operated on the Yeh and Shum morphometric tabulated data; a better result is obtained with MPPD adopting a different bronchial tree (PNNL) and the KH original method. The correction/rescaling of the morphometric parameters based on the lung function (functional residual capacity) does not necessarily lead to better results; see the last two columns with the *F* subscript. A detailed analysis of why our prediction is better than others requires the entry into the very intimate details of the different algorithms and is beyond the scope of this first introductory paper.

### 3.2. Regional Deposition

With the analysis on the total deposition fraction, carried out in the previous section, we have demonstrated that our algorithm is capable of correctly predicting the amount of aerosol dose deposited and exhaled by the subjects. A slightly more challenging task is to predict the regional deposition, i.e., distinguishing how much of the deposited dose lands in the extra-thoracic and conducting airways ($a_{i}=0$, TB deposition fraction) or in the functional airways ($a_{i}>0$, pulmonary deposition fraction).

**Figure 9:**
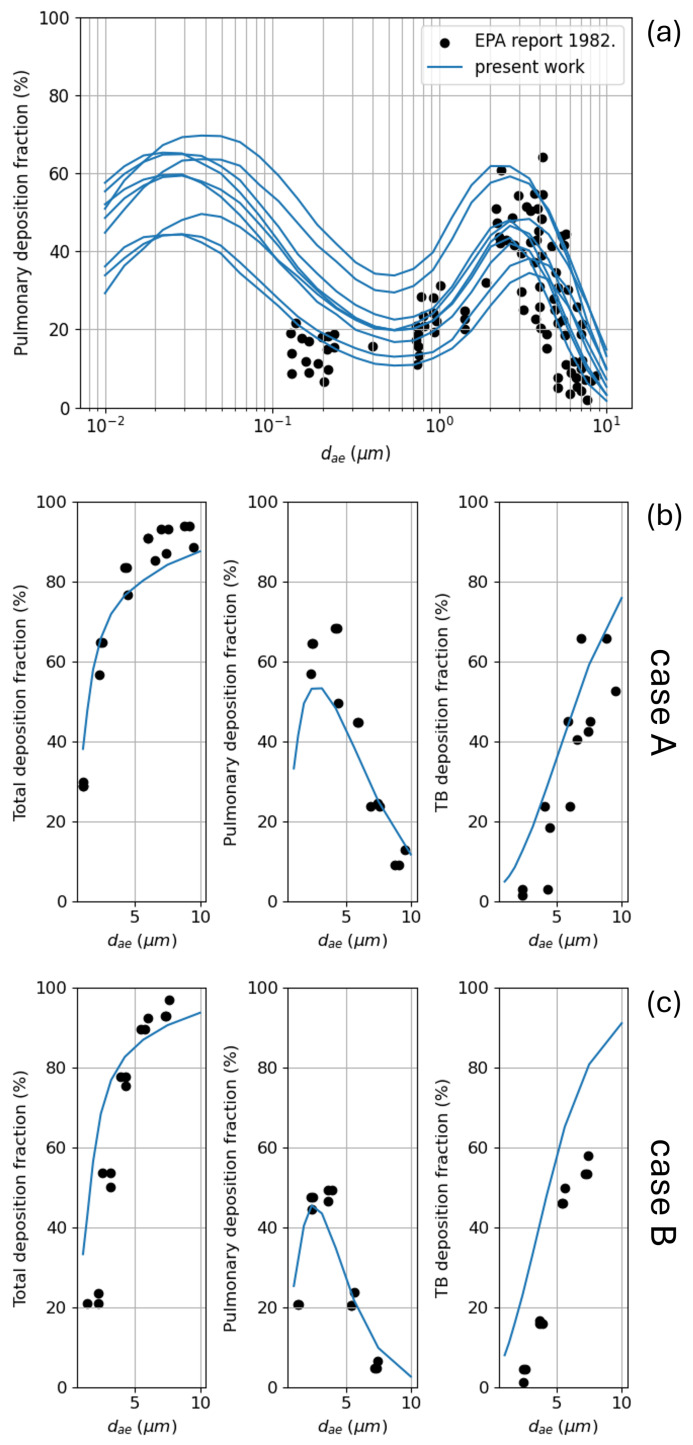
(**a**) Pulmonary deposition fraction as a function of the aerosol aerodynamic particle/droplet diameter for the dataset of reference [60] (black dots) and simulation prediction (blue line). (**b**,**c**) Total, pulmonary, and tracheobronchial deposition fractions as a function of the aerosol aerodynamic particle/droplet diameter for the dataset of reference [61] (black dots) and simulation prediction (blue line).

A rich dataset for pulmonary deposition as a function of the aerosol particle/droplet size is available in the 1982 US Environmental Protection Agency [60]; the data are plotted as black dots in Figure 9a. As for total deposition, combining the tabulated data for minimum, mean, and maximum values of $V_{tidal}$ (500, 1055, and 1500 mL) and *T* (3.3, 4.4, and 8.6 s), we computed the nine possible deposition curves shown in the same panel. Again, the agreement with the measured data is quite good in both the average values and in the inter-subject variability. One of the oldest datasets available for regional deposition has been provided by Stahlhofen et al. [61] for two subjects named case A ($V_{tidal}=1$ L and T=8 s) and case B ($V_{tidal}=1.5$ L and T=4 s); the data for total, pulmonary, and tracheobronchial deposition are reported as black dots in panels (b) and (c) of Figure 9. Also, here the simulation predictions (blue curves) match nicely with the shape of the profiles in both the position and the height of the maximum for pulmonary deposition, and in the slope for the monotonic total and tracheobronchial deposition.

### 3.3. Lobar and Sub-Lobar Deposition

**Figure 10:**
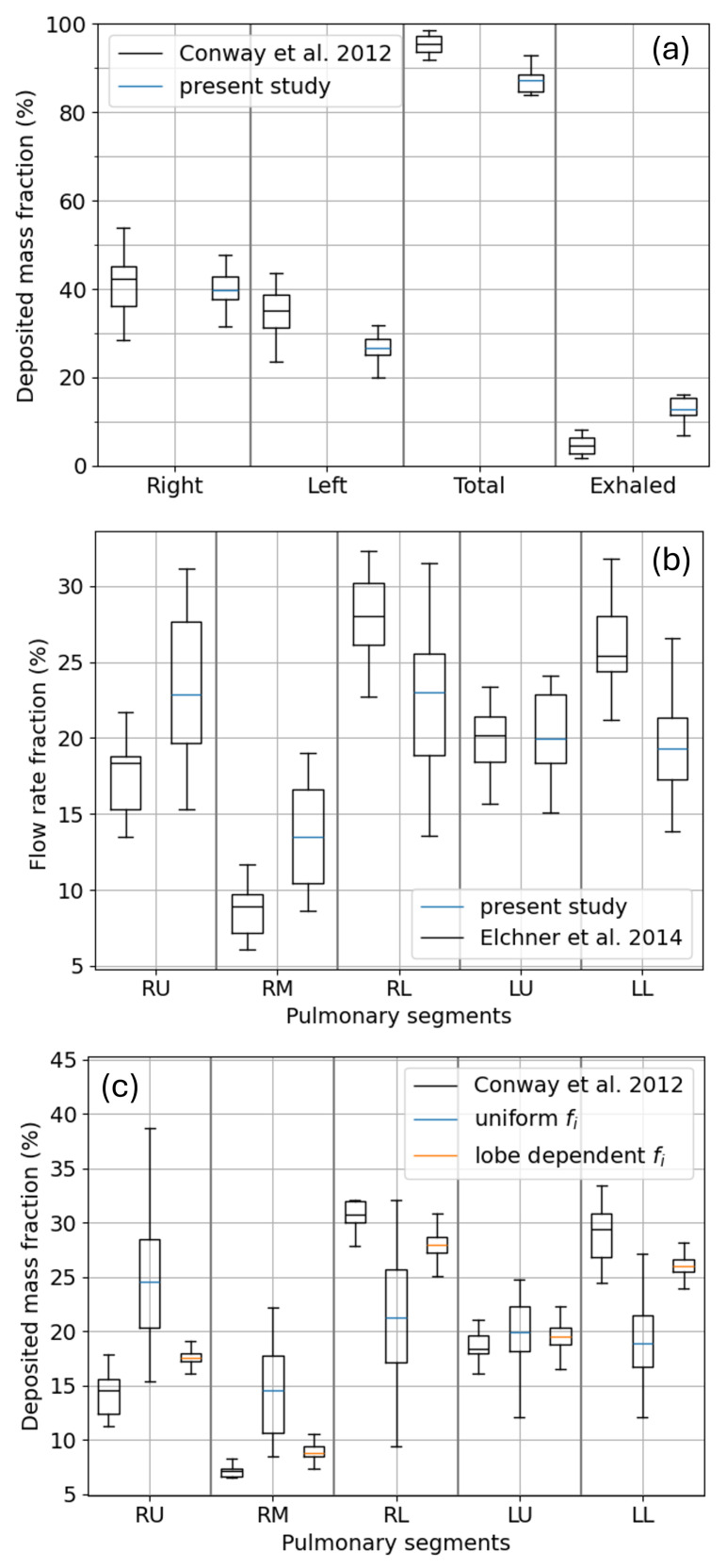
(**a**) Box plot for the lobar aerosol deposition comparing the experimental dataset by Conway et al. (black) [62] and the predictions of our model (blue). (**b**) Box plot for the airflow rate fraction entering in each of the lung sub-lobes comparing the reference dataset by Elchner et al. (black) [45] and the prediction of our model (blue). (**c**) Box plot for the sub-lobar aerosol deposition comparing the experimental dataset by Conway et al. (black) [63] and the prediction of our model in the presence of uniform (blue) and non-uniform (orange) lobe ventilation. The statistics on our model results are obtained by simulating 20 different stochastically generated trees, each of them with the 22 profiles reported by Conway et al. [62].

Concerning total and regional deposition, the Monte Carlo model predictions are known to be insensitive to the detailed description of the airflow splitting in the proximal bifurcations [13]. On the contrary a correct estimation of the lobar or sub-lobar deposition requires the airflow splitting to be as close as possible to the realistic, physiological one. A lobar deposition study is described in the work by Conway et al. [62]. The exhaled mass, the deposited one, as well as the fraction deposited in the left and right lobes are shown in black in the box plot of Figure 10a. The linear breathing profiles measured for the different subjects are tabulated in their paper together with the aerosol size distribution, which in this case is a log-normal distribution rather than the uniform one adopted in the rest of this work. To compare our model prediction with the measured data, we run a set of simulations for each recorded inhalation profile over 20 stochastically generated trees; thus, the data plotted in blue in the box plot of panel (a) include both the variability due to different inhalation profiles and different tree geometries. The agreement with respect to the measured data is satisfactory and is tightly connected to the model’s ability to correctly capture the left–right flow splitting in the first bifurcation, ensured using Equation (10) for the splitting coefficients $s_{i}$. In fact, using Equation (4), which promotes a 50–50% splitting at each bifurcation, also results in 50–50% left–right deposition. As airflow splitting seems to play a major role in determining local deposition, before looking at the sub-lobar deposition, one should check if Equation (10) is able to reproduce the correct partition of air among the five sub-lobes (as defined in Figure 1). The results for the simulated airflow splitting are shown in the box plot of Figure 10b, averaged over 20 stochastically generated trees, and compared to the measurements by Elchner et al. [45] obtained by averaging over 16 subjects. Qualitatively the anisotropy in the airflow distribution is correctly captured; however, from a quantitative point of view, an underestimation is clearly visible for RL and LL, compensated by an overestimation in RU and RM. A proportional quantitative mismatch is found for the sub-lobar deposition, as shown by the blue box plot in panel (c).The reference sub-lobar deposition dataset is reported in the paper by Grill et al. [63] and, according to the authors, is obtained from the experimental data by Conway et al. [62] by averaging 22 deposition experiments over 11 subjects. A sub-lobar deposition calculation is performed by Winkler-Heil et al. [64], through a KH Monte Carlo method, and is found to be in good agreement with the Conway dataset. They implemented an empirical, inhomogeneous, and asynchronous ventilation model as well as morphometric statistical distributions differentiated for each sub-lobe for the construction of their bronchial trees. From the results they present, it seems the major role, in matching the sub-lobar deposition, is played by the differential morphometric statistics rather than by the ventilation model. On our side, an attempt to improve the ventilation anisotropy of panel (b) can be made by modulating the alveolar density in the sub-lobes or by differentiating their efficiency in recalling air, i.e., redistributing the $a_{i}$ or the $f_{i}$ values, respectively, in Equation (8). For the sake of brevity and simplicity, we act on the $f_{i}$ values only, although it is plausible that both $a_{i}$ and $f_{i}$ can be differently distributed among the sub-lobes. Moreover, as we want to model a different propensity of each sub-lobe to inflate and deflate as a whole, we further assume that $f_{i}$ has the same values within each sub-lobe. We thus switch from a homogeneous ventilation, expressed by the following condition:

(16) $$f_{i}=f_{RU}=f_{RM}=f_{RL}=f_{LU}=f_{LL}=1,$$

to the set of values $f_{RU}=0.29$, $f_{RM}=0.29$, $f_{RL}=3.78$, $f_{LU}=0.52$, and $f_{LL}=1.12$ that, used with the flow splitting Equation (4), allows to impose the flow splitting reported by Elchner. The resulting sub-lobar deposition, shown in the orange box plot in Figure 10c, is now in good agreement with the Conway and Winkler-Heil data. A similar result can be achieved through Equation (10) but requires a bit more algebra, or the implementation of a recursive procedure, to compute the $f_{i}$ values that are able to impose the Elchner splitting. Now, in fact, part of the anisotropy is already induced by the different diameter of the ducts. An alternative path leading to a physiological flow splitting among the sub-lobes has been proposed by Phillips and Kaye [65] and consists of adopting different power laws for the duct diameters $D_{i}$ directly within the flow splitting Equation (4).

To conclude, lobar, sub-lobar, and local deposition are extremely sensitive to the airflow splitting coefficient at bifurcations. If the correct flow splitting is directly achieved with mechanical ventilation models or imposed through empirical ones, the correct aerosol deposition is obtained. Whether the ventilation anisotropy among the sub-lobes is more impacted by a different local morphology of the airways or by a different elasticity of the parenchyma or simply by a different availability of space for stretching during inhalation remains to be investigated further.

## 4. Conclusions and Future Developments

We have presented an evolution of the original Monte Carlo algorithm proposed by KH to predict aerosol deposition in healthy human lungs. The new implementation enables working with pre-constructed bronchial trees generated with different algorithms and software and is possibly based on patient CT-scans. We tested the code predictions for total, regional, lobar, and sub-lobar deposition against in vivo published datasets. The agreement between simulations and experimental data is remarkable in both the average values and the variability associated with inter-subject differences, e.g., different inhalation profiles. The trends for the deposited fraction as a function of the aerosol size and the flow rate intensity are quantitatively reproduced. An even better agreement is expected when the model predictions are carried out on the specific bronchial trees of the subjects involved in the deposition experiments, relying on old datasets for which the CT-scans of the involved subject are not available; such a possibility remains out of reach at present. Notably, our model adheres to the experimental data without making use of calibration or correction factors such as the *enhancement factor* or the *mixing factor* introduced by KH [17,66]. The code predictivity could be challenged further if subject-specific local deposition data were available (e.g., coming from a 2D or 3D scintigraphy) and accompanied by the subject CT-scan from which the tree morphology could be extracted.

The results on lobar and sub-lobar deposition presented in Section 3.3 suggest that reproducing a correct flow splitting through the bifurcation is crucial to obtain the correct quantitative local deposition. To this aim, the model could be improved significantly by replacing the empirical flow splitting coefficients, calculated through Equation (10), with a more realistic mechanical breathing model. Possibly, one whose parameters can be related to the lung function data typically measured on patients through spirometry, e.g., FEV1 and FVC.

Even without a mechanical breathing model already implemented, disease models can be applied to the pre-constructed trees to see how different diseases and different severities can alter the drug delivery. Bronchoconstriction and bronchiectasis can be modeled by modifying the duct diameter $D_{i}$ according to certain rules and with a frequency related to the disease severity. Loss of lung function, typical of pulmonary fibrosis or emphysema, can be modeled by modifying the alveolation index $a_{i}$ or the functional factor $f_{i}$ distributions. In both cases Equation (10) should grant a significant readjustment of the airflow distribution in the whole tree with consequences on local aerosol deposition. In this respect, studies on large datasets of labeled medical images are necessary to collect some statistical information on the morphometric and functional alteration the trees undergo due to the different disease and their severity.

From the aerosol physics point of view, several points remain open, deserving further investigation. These concern the impact on aerosol deposition of the following: (i) the different growth models for the bronchial trees; (ii) the choice of the equation for the joint probability $P_{i}$; and (iii) the different particle splitting mechanism at bifurcations. Other questions more related to aerosol delivery and product design concern the impact on aerosol deposition of the following: (i) inter-subject variability in healthy volunteers; (ii) disease severity and presence of co-morbidities; and (iii) different inhalation indications, different inhalation maneuvers, misuse, and mistakes during the inhalation maneuver.

In a long term perspective, the critical analysis we started in this first work is an important step towards the realization of a multi-scale model combining CFD simulations to treat dose aerosolization inside the inhaler [67,68,69,70,71] and deposition in extra-thoracic and upper airways, and Monte Carlo methods to cover deposition in the distal airways. Besides favoring inhaler design optimization and drug delivery efficiency, the quantitative predictions of such a multi-scale model could be used to perform physiologically based pharmacokinetics studies [72,73]. Lastly, the same type of approach could be easily extended to improving pre-clinical toxicology animal models, hastening pharmacology studies and their translation to humans [74].

## Figures and Tables

**Figure A1:**
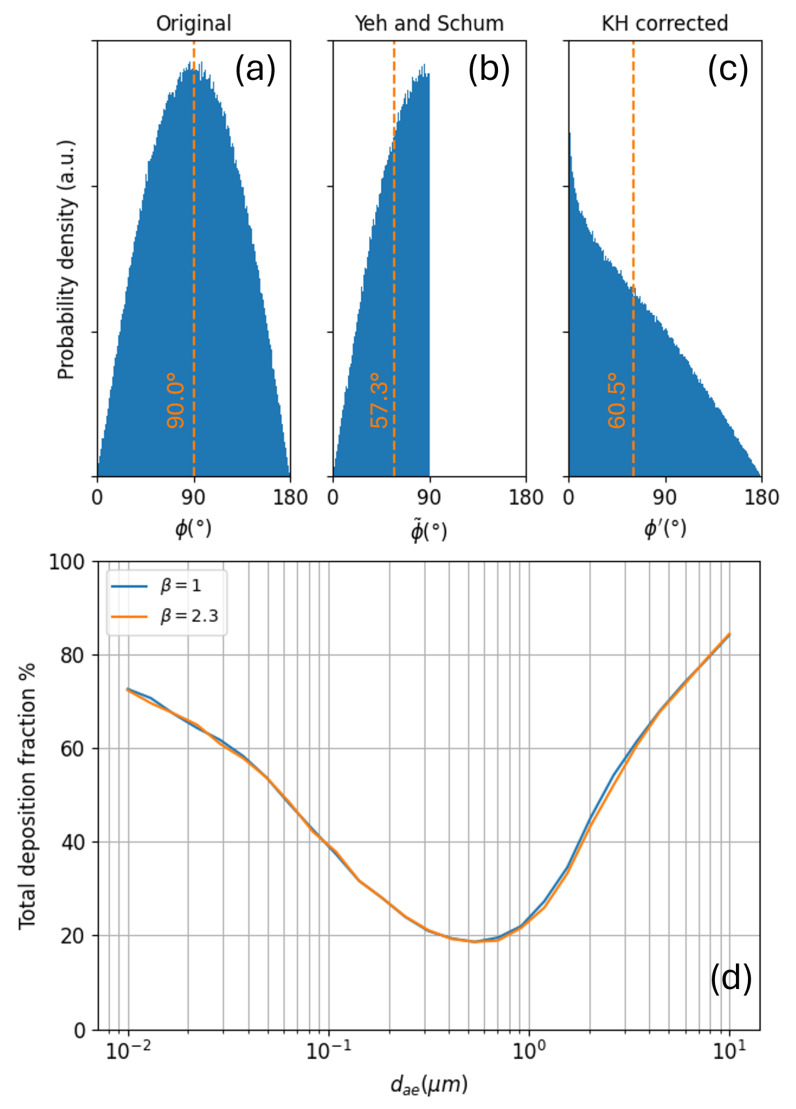
Histograms of the gravity angle distribution for the 20th generation, for (**a**) the original $\phi_{i}$ distribution, (**b**) the back-folding transformation of Equation (A6), and (**c**) the KH correction of Equation (A5). (**d**) Total aerosol deposition fraction calculated for the trees with and without the KH correction, the breathing conditions, and the model parameters are the same adopted in the calculations of Section 2.3.5.

## Data Availability

The data presented in this article as well as the code designed to produce them belong to Chiesi Farmaceutici. They can be made available upon request. The access is subject to approval and to a data sharing agreement.

## Appendix A. Indexes Relations

Assuming dichotomous branching and the duct indexing convention illustrated in Figure 1c, several algebraic relations can be easily derived between parent and children ducts and between ducts and generation number. If the bronchial tree has *N* generations, the total number of ducts $N_{tot}$ can be computed as follows:

(A1) $$N_{tot}=2^{N+1}-1,$$

while the number of bifurcations N as follows:

(A2) $$N=2^{N}-1.$$

Given a duct of index *i*, the generation *z* to which it belongs can be computed as follows:

(A3) $$z=floor[\log_{2}\left(i+1\right)].$$

The first and last duct indexes of generation *z* are $2^{z}-1$ and $2^{z+1}-2$, respectively. If a parent duct has index *k*, its two children have index 2k+1 and 2k+2; conversely, if a child duct has index *s*, its parent has index *k*:

(A4) $$k=\left\{\begin{array}{c} \frac{s-2}{2}\;s\;even\\ \frac{s-1}{2}\;s\;odd. \end{array}\right.$$

## Appendix B. Details on KH-like Tree Generation

In their morphometric study of an acinus, Haefeli-Bleuer and Weibel estimated an average of ≃445 alveolar sacs per acinus. Multiplying for the smallest number of acini proposed by KH (20 thousands) gives a total of 8.9 millions of alveolar sacs, i.e., a number slightly larger than the total number of ducts available in the 23rd generation (i.e., $2^{23}$). It is thus clear that forcing the tree to have a maximum of 23 generations and simultaneously allowing a premature capping of the airways (already at generations 21 and 22) will lead to a maximum number of alveolar sacs much smaller than the correct value. This apparent inconsistency originates from the fact that real bronchial trees can extend beyond generation 23 and are only *on average* terminated at generation 23 [38]. Thus, even if some airway is prematurely capped at generation 21, some other airway can compensate by reaching generation 24 or 25, increasing the number of ducts (and thus of terminal alveolar sacs) and allowing us to reach the measured value of 8.9 millions sacs. Extending the tree far above generation 23 significantly enhances the computational cost to both generate the tree and to perform deposition simulations. On the other hand, matching the correct number of alveolar sacs is important to obtain quantitative deposition predictions. A good compromise is represented by maintaining 23 generations as the maximum tree extension but not to force premature capping, i.e., all the airways are cupped at generation 23 so that the total number of sacs is very close to the value proposed by Haefeli-Bleuer and Weibel.

In their first work on aerosol deposition, KH [17] noticed that by averaging the gravity angle over all the ducts of each distal generation, a value of ≃90° was obtained. This was apparently contradicting the finding by Yeh and Schum [14] reporting a value of $60^{\circ }$. They concluded that in physiological conditions there must be an asymmetry in the duct orientation with the downward direction prevailing over the upward one. To account for this asymmetry, they proposed a norm-conserving rotation operation to be applied to each duct versor, modifying the gravity angle $\phi_{i}$ thus biasing downward its generation-averaged value:

(A5) $$\begin{array}{cc} n_{i}^{z\prime }=2{\left(\frac{n_{i}^{z}+1}{2}\right)}^{\beta }-1 & \\ n_{i}^{x\prime }=n_{i}^{x}{\left(\frac{1-{\left(n_{i}^{z\prime }\right)}^{2}}{1-{\left(n_{i}^{z}\right)}^{2}}\right)}^{1/2}\;n_{i}^{y\prime }=n_{i}^{y} & {\left(\frac{1-{\left(n_{i}^{z\prime }\right)}^{2}}{1-{\left(n_{i}^{z}\right)}^{2}}\right)}^{1/2} \end{array}$$

where $\hat{n_{i}}=\left(n_{i}^{x},n_{i}^{y},n_{i}^{z}\right)$ and ${\hat{n_{i}}}^{\prime }=\left(n_{i}^{x\prime },n_{i}^{y\prime },n_{i}^{z\prime }\right)$ are the original and corrected duct versors, respectively, and β≥1 is the correction magnitude (β=1 means no correction). In our bronchial trees we observe the same average gravity angle. The distribution of $\phi_{i}$ in the 20th generation, plotted in Figure A1a, confirms that the value comes from a perfect symmetry between upward and downward oriented ducts. A symmetric distribution has been reported by Monfared et al. [75] for the central airways by analyzing 10 CT-scans of patients of different sexes and ages. The explanation for this apparent inconsistency is in the way Yeh et al. [76] defined their experimentally measured gravity angle, implying a transformation that reduces from $\left[0^{\circ },180^{\circ }\right]$ to $\left[0^{\circ },90^{\circ }\right]$ is the interval of variation of $\phi_{i}$. More precisely, their reduced gravity angle $\tilde{\phi_{i}}$ is obtained through the following transformation:

(A6) $$\tilde{\phi_{i}}=\left\{\begin{array}{cc} \phi_{i} & if\;\phi_{i}<90^{\circ }\\ 180^{\circ }-\phi_{i} & if\;\phi_{i}\geq 90^{\circ }, \end{array}\right.$$

which back-folds upward ducts into downward ones; the resulting distribution is shown in panel (b) of Figure A1. Averaging over this back-folded distribution, one gets the $60^{\circ }$ result, which, however, represents a tree with perfectly symmetric orientation of ducts with respect to gravity. On the contrary, if the orientation correction of Equation (A5) is applied to $\phi_{i}$, a value of β=2.3 is necessary to achieve an average of $60^{\circ }$ and the asymmetrical distribution is shown in panel (c) of Figure A1. KH report a value β=1.35; however, their correction of $\phi_{i}$ is made *on-the-fly* as the individual airway paths are constructed for each individual particle trajectory. In our case the tree is first generated entirely and then the $\phi_{i}$ correction is applied. This major difference is likely to be the responsible for the different β value. Fortunately particle deposition seems to be almost insensitive to the correction of the gravity angle, as visible in Figure A1d, where the same deposition simulation detailed in Section 2.3.5 is performed with both β=1 (no correction applied) and β=2.3. Based on the considerations presented in this section, the gravity angle correction has been disregarded throughout our work.

## Appendix C. Time Integration of the Aerosol Particle Trajectory

Adopting Equation (13) to calculate the particle trajectory and its crossing time $\tau_{i}$ when $L_{i}/U_{i}>\Delta t$ implies two assumptions: (i) the particle motion is purely rectilinear along the duct main axis; and (ii) the particle velocity coincides with the air velocity. This latter point is surely true for small size or small density particles in the distal airways; in fact, these conditions lead to small relaxation time and Stokes number, ensuring the particle will follow the air stream with little to no inertia. It is more questionable for large particles at small $Q_{0}\left(t\right)$ values, i.e., close to a switching point between inhalation and exhalation phases in a tidal profile. In any case, although questionable, the assumption that particles of any diameter always move at the same air velocity $U_{i}$ is coherent with the rest of the mode. In fact, such an assumption is at the basis of all the derivations of deposition probability equations used in any Monte Carlo method.

Discretized through an implicit Euler scheme, Equation (13) reads as follows:

(A7) $$\begin{array}{cc} x(t+\Delta t) & =x\left(t\right)+\Delta t\;U_{i}\left(t+\Delta t\right)\\ \; & =x\left(t\right)+\frac{4S_{i}\Delta t}{\pi D_{i}^{2}}Q_{0}\left(t+\Delta t\right). \end{array}\;$$

As $Q_{0}\left(t\right)$ changes its sign, the particle can travel back and forth along the duct until $x\left(t+\Delta t\right)=L_{i}$ during an inhalation phase or x(t+Δt)=0 during an exhalation phase. Labeling with *n* the number of time-steps it takes to reach one of these two conditions, the general definition of the crossing time $\tau_{i}$ becomes the following:

(A8) $$\tau_{i}=\left\{\begin{array}{cc} L_{i}/U_{i} & if\;L_{i}/U_{i}\leq \Delta t\\ n\;\Delta t & if\;L_{i}/U_{i}>\Delta t. \end{array}\right.$$

While for the velocity $U_{i}$, which is needed in the probability equations, our modified model prescribes the following:

(A9) $$U_{i}=\left\{\begin{array}{cc} \frac{4S_{i}}{\pi D_{i}^{2}}Q_{0} & if\;L_{i}/U_{i}\leq \Delta t\\ \frac{1}{n}\sum_{k=1}^{n}\left|U_{i}\left(k\;\Delta t\right)\right| & if\;L_{i}/U_{i}>\Delta t, \end{array}\right.$$

i.e., the average of the absolute value of all the air velocities the particle has been subject too while crossing the duct.

## Appendix D. Detailed Implementation of Deposition Probability Equations

This appendix is devoted to the detailed description of the deposition probability equations implemented in our core. Let us start with the inertial impaction probability $I_{i}$ taken from the Yeh and Schum [14] work:

(A10) $$I_{i}=1-\frac{2}{\pi }arccos\left(\theta_{i}\;Stk_{i}\right)+\frac{1}{\pi }sin\left(2\;arccos\left(\theta_{i}\;Stk_{i}\right)\right),$$

where the Stokes number $Stk_{i}$ of the *i*-th duct is computed as follows:

(A11) $$Stk_{i}=C_{c}\;\frac{\rho d^{2}}{18\mu }\;\frac{U_{i}}{D_{i}},$$

with $U_{i}$ fluid velocity from Equation (A9) and $C_{c}$ the Cunningham correction factor defined as

(A12) $$C_{c}=1+\frac{2\lambda }{d}\left(a_{1}+a_{2}e^{-a_{3}d/\lambda }\right),$$

where $\lambda =7.2\cdot 10^{-8}$ m is the mean free path of air molecules, and the coefficients $a_{1}=1.257$, $a_{2}=0.4$, and $a_{3}=0.55$ are taken from reference [77]. For the trachea, one has $\theta_{0}=0$ so that $I_{0}=0$; thus, the impaction deposition at the first bifurcation is accounted only by the two trachea children ducts (duct index 1 and 2). For $Stk_{i}≃1$ deposition is mainly determined by the bifurcation angle and is maximized, $I_{i}\to 1$, if the bifurcation has an elbow shape, i.e., $\theta_{i}≃\pi /2$.

According to Yeh and Schum [14], in the well-mixed aerosol hypothesis, the probability for gravitational settling $G_{i}$ in a duct during all breathing phases can be computed as follows:

(A13) $$G_{i}=1-e^{-\frac{16}{3\pi }\kappa },\;$$

where $\kappa =3/4v_{settling}\;t/D_{i}\;\left|sin\left(\phi_{i}\right)\right|$ contains the aerosol settling velocity $v_{settling}=C_{c}\;\rho d^{2}g/18\mu$, g is the gravity acceleration, $\phi_{i}$ the gravity angle, and *t* is a characteristic time calculated through Equation (A8) during inhalation and exhalation phases or replaced by $\Delta T_{bh}$ during the breath-holding phases. For perfectly vertical pipes gravitational settling is not supposed to contribute to aerosol deposition; in fact, for $\phi_{i}=0$ one has κ=0 and thus $G_{i}=0$ as well. On the contrary it is supposed to promote deposition of heavy or large particles. Indeed, these give rise to high $v_{settling}$ values, leading to high κ and $G_{i}$. Finally, gravitational settling is supposed to dominate deposition in the distal airways, where the duct diameter is small. This aspect is also captured by the probability equation as κ is inversely proportional to $D_{i}$. The well-mixed aerosol hypothesis assumes turbulence and secondary flows in the proximal airways are efficiently dispersing the aerosol uniformly along the duct cross section; other formulations exist for Poiseuille and plug flows [46]. For aerosol particles in alveoli KH [17] propose the following equation:

(A14) $$G_{i}=\left\{\begin{array}{cc} \frac{1}{2}\frac{v_{settling}t}{D_{i}}\left[3-{\left(\frac{v_{settling}t}{D_{i}}\right)}^{2}\right] & if\;\frac{v_{settling}t}{D_{i}}<1\\ 1 & if\;\frac{v_{settling}t}{D_{i}}\geq 1, \end{array}\right.$$

where *t* is the permanence time of the aerosol particle/droplet in the alveolus.

For the deposition probability due to Brownian diffusion $B_{i}$ in ducts during inhalation and exhalation phases, Yeh and Schum [14] adopted the following equation (in laminar regime):

(A15) $$B_{i}=\left\{\begin{array}{cc} 1-0.819e^{-14.63\Delta }-0.0967e^{-89.22\Delta }-0.0325e^{-228\Delta }-0.0509e^{-125.9\Delta^{2/3}} & if\;\Delta <0.16853\\ 1 & if\;\Delta \geq 0.16853, \end{array}\right.$$

with $\Delta =D_{c}\tau_{i}/D_{i}^{2}$ where $D_{c}$ is the particle diffusion coefficient in air $D_{c}=k_{b}TC_{c}/3\pi \mu d$, $k_{b}$ is the Boltzmann constant, and *T* the temperature (37 °C). Alternative formulations exist to account for turbulent flows [46]. During breath-holding the previous equation must be substituted by the following:

(A16) $$B_{i}=1-e^{-23.136\Delta },$$

now Δ is computed by replacing the duct crossing time $\tau_{i}$ with the breath-holding time $\Delta T_{bh}$. Finally for aerosol depostion in alveoli, the probability equation becomes the following:

(A17) $$B_{i}=1-\frac{6}{\pi^{2}}\sum_{n=1}^{\infty }\frac{e^{-4D_{c}\pi^{2}n^{2}t/D_{i}^{2}}}{n^{2}},$$

where *t* is now the total residence time of the aerosol particle/droplet in the alveolus. We tested the sub-sum of the series above. Finding a good convergence already for n=500, we thus set n=1000 for all the calculations presented in this paper. In all the equations for $B_{i}$ the geometry and particle properties appear only through the quantity Δ. It is thus easy to see that Brownian deposition is more effective for small particles and high temperatures; in fact, in both cases $D_{c}$ gets large, leading to $B_{i}\to 1$. As for gravitational settling Brownian diffusion is also expected to dominate deposition in small distal airways. In the probability equation this is ensured but with the inverse proportionality between Δ and $D_{i}$.

## Appendix E. List of Symbols Adopted in the Paper

This appendix contains a summary of the symbols adopted throughout the paper. For the sake of clarity they have been spitted into three tables containing, respectively, those related to the bronchial tree properties (Table A1), those describing the air properties (Table A2), and those listing aerosol/particle properties (Table A3).

**Table A1:** List of symbols related to the bronchial tree geometry.

Symbol	Meaning
*N*	number of generations composing the bronchial tree
$N_{tot}$	number of ducts composing the bronchial tree
N	number of bifurcations composing the bronchial tree
${\hat{n}}_{i}$	versor indicating the *i*-th duct direction
$D_{i}$	average diameter of the *i*-th duct
$L_{i}$	length of the *i*-th duct
$\theta_{i}$	branching angle of the *i*-th duct
	(angle between ${\hat{n}}_{i}$ and its parent versor ${\hat{n}}_{p}$)
$\Theta_{i}$	bifurcation angle of the *i*-th duct
	(angle between its two child duct versor ${\hat{n}}_{c}$ and ${\hat{n}}_{c+1}$)
$\phi_{i}$	gravity angle of the *i*-th duct
	(angle between the gravity versor $\hat{g}$ and ${\hat{n}}_{i}$)
$\varphi_{i}$	rotation angle of the *i*-th duct
	(angle between the plane containing *i*-th and (i+1)-th ducts
	and the one containing the *i*-th children)
$\delta_{i}$	non-planarity angle of the *i*-th duct
	(angle between the plane containing the *i*-th duct and the one
	containing its two children)
$a_{i}$	alveolation index of the *i*-th duct

**Table A2:** List of symbols related to the airflow in the bronchial tree.

Symbol	Meaning
$V_{tot}$	total lung volume
$V_{ducts}$	volume of the cylindrical ducts
$V_{alv}$	total alveolar volume
$V_{tidal}$	tidal volume
$U_{i}$	average air velocity in the *i*-th duct
$Q_{i}$	airflow rate in the *i*-th duct
$Q_{0}$	airflow rate at the mouth and trachea (i=0)
$s_{i}$	splitting coefficient of the *i*-th duct
$S_{i}$	cumulative splitting coefficient of the *i*-th duct
$\rho_{air}$	air density at 37 °C
μ	air dynamic viscosity at 37 °C

**Table A3:** List of symbols related to the aerosol properties.

Symbol	Meaning
*d*	diameter of aerosol particle/droplet
$d_{ae}$	aerodynamic diameter of aerosol particle/droplet
ρ	density of the aerosol particle/droplets
$P_{N}\left(d\right)$	aerosol particle/droplet size number distribution
$P_{M}\left(d\right)$	aerosol particle/droplet size mass distribution
$T_{tot}$	total duration of the simulation deposition
$\Delta T_{i}$	duration of inhalation phase
$\Delta T_{bh}$	duration of breath-holding phase
$\Delta T_{e}$	duration of exhalation phase
*T*	period of the tidal breathing sinusoid
$V_{tidal}$	air volume inhaled and exhaled during a tidal period
$T_{start}$	time instant of particle/droplet insertion
$\Delta T_{ins}$	time interval for aerosol insertion
τ	particle/droplet flight time
$\tau_{i}$	particle/droplet crossing time for the *i*-th duct
$I_{i}$	inertial impaction deposition probability in the *i*-th duct
$G_{i}$	gravitational settling deposition probability in the *i*-th duct
$B_{i}$	Brownian diffusion deposition probability in the *i*-th duct
$P_{i}$	joint probability to survive deposition in the *i*-th duct
